# Photo(electro)catalytic Water Splitting for Hydrogen Production: Mechanism, Design, Optimization, and Economy

**DOI:** 10.3390/molecules30030630

**Published:** 2025-01-31

**Authors:** Xingpeng Li, Chenxi Zhang, Jiafeng Geng, Shichao Zong, Pengqian Wang

**Affiliations:** 1 Key Laboratory of Subsurface Hydrology and Ecological Effects in Arid Region, Ministry of Education, School of Water and Environment, Chang’an University, Xi’an 710064, China; lxp990617@163.com (X.L.); zhuo782713lanl@163.com (C.Z.); gengjf@chd.edu.cn (J.G.); 2 Key Laboratory of Eco-Hydrology and Water Security in Arid and Semi-Arid Regions of the Ministry of Water Resources, School of Water and Environment, Chang’an University, Xi’an 710064, China

**Keywords:** photocatalysis, electrocatalysis, water splitting, hydrogen production

## Abstract

As an energy carrier characterized by its high energy density and eco-friendliness, hydrogen holds a pivotal position in energy transition. This paper elaborates on the scientific foundations and recent progress of photo- and electro-catalytic water splitting, including the corresponding mechanism, material design and optimization, and the economy of hydrogen production. It systematically reviews the research progress in photo(electro)catalytic materials, including oxides, sulfides, nitrides, noble metals, non-noble metal, and some novel photocatalysts and provides an in-depth analysis of strategies for optimizing these materials through material design, component adjustment, and surface modification. In particular, it is pointed out that nanostructure regulation, dimensional engineering, defect introduction, doping, alloying, and surface functionalization can remarkably improve the catalyst performance. The importance of adjusting reaction conditions, such as pH and the addition of sacrificial agents, to boost catalytic efficiency is also discussed, along with a comparison of the cost-effectiveness of different hydrogen production technologies. Despite the significant scientific advancements made in photo(electro)catalytic water splitting technology, this paper also highlights the challenges faced by this field, including the development of more efficient and stable photo(electro)catalysts, the improvement of system energy conversion efficiency, cost reduction, the promotion of technology industrialization, and addressing environmental issues.

## 1. Introduction

**Figure 1:**
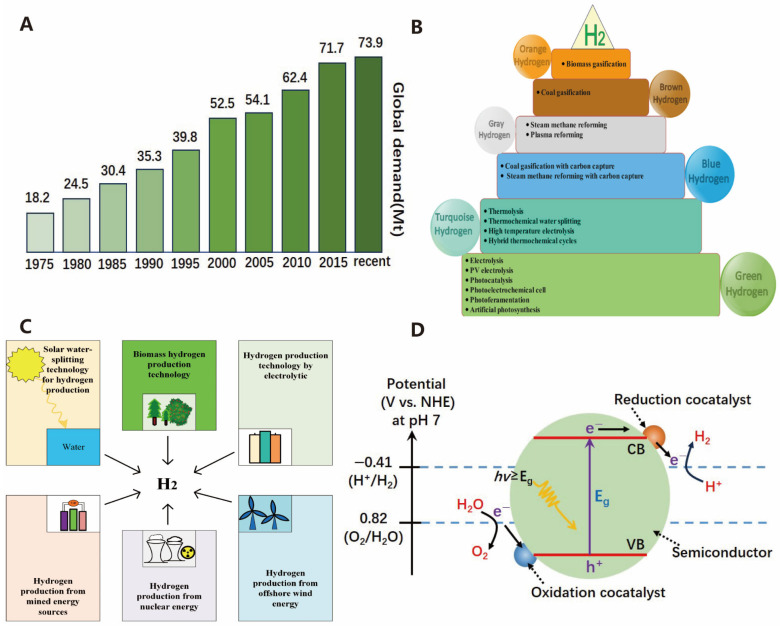
(**A**) Global demand for pure hydrogen. (**B**) Hydrogen production methods with their color codes (adapted from [4] with permission from Elsevier, copyright 2024). (**C**) Different green hydrogen production technologies (reproduced from [7] with permission). (**D**) Schematic diagram of the reaction of water splitting on a semiconductor photocatalyst (adapted from [8] with permission from Elsevier, copyright 2018).

Currently, driven by the increasing prosperity and growth of emerging economies, global energy demand continues to rise [1]. However, fossil fuels still dominate the global energy consumption structure. The combination of these two factors has further led to the over-exploitation of fossil fuels and subsequent pollution issues. Therefore, the development of clean and renewable energy sources is an urgent need for sustainable human development in the future [2]. There are many cleaner and more sustainable alternatives to traditional fuel resources, such as wind energy, solar energy, hydropower, and photovoltaics. However, each alternative has its limitations, making it more challenging to distinguish themselves from traditional fuels [3]. As shown in Figure 1A, hydrogen is increasingly gaining a favorable foothold in the advancement of the energy sector owing to its excellent properties of being pollution-free, a high calorific value, and reusable [4]. Reducing the reliance on fossil fuels as raw materials and developing new technologies for hydrogen production are among the goals of the hydrogen energy industry. As shown in Figure 1B,C, currently, widely researched novel hydrogen production technologies include electrocatalytic hydrogen production, photocatalytic hydrogen production, and bioenergy-based hydrogen production, among others [5]. With the gradual maturity of hydrogen production technologies, hydrogen energy is expected to be produced and utilized on a large scale in the future. Therefore, researching hydrogen production technologies, especially green hydrogen production technologies, holds great potential in addressing energy crises and environmental issues. The optimal source of green hydrogen is water splitting achieved through electrocatalytic and photocatalytic means. Water splitting for hydrogen production offers numerous advantages, but the hydrogen evolution reaction (HER) and oxygen evolution reaction (OER) are inherently slow, especially the OER, thus requiring efficient catalysts to overcome kinetic barriers and promote a faster and more effective water splitting process [6].

In 1972, Fujishima and Honda first discovered that photocatalytic reactions could occur using a titanium dioxide (TiO_2_) semiconductor single-crystal electrode, decomposing water into hydrogen and oxygen [9]. This discovery marked the birth of photo(electro)catalytic water splitting technology and laid the foundation for subsequent research. Initially, the primary photocatalyst was titanium dioxide (TiO_2_); however, its application in photocatalysis and electrochemistry was limited due to disadvantages such as high electron–hole recombination rates, low electron mobility, poor conductivity, and small reversible capacity [10]. As research progressed, scientists began to seek better photocatalyst materials. They found that certain transition metal compounds [11] and metal oxides [12] exhibited good catalytic activity. These materials have suitable band structures, enabling them to effectively absorb solar energy and promote water decomposition reactions. In recent years, scientists have developed various novel photocatalysts, such as semiconductor nanomaterials, MOFs (Metal–Organic Frameworks), COFs (Covalent Organic Frameworks), and others [13]. These materials possess better light absorption properties and catalytic activity, enabling efficient photo(electro)catalytic water splitting reactions. Additionally, by employing nanotechnology and surface modification techniques, the stability and catalytic efficiency of photocatalysts can be further enhanced [14]. Through the regulation of material structure and surface properties, the efficient utilization of photocatalysts can be achieved [15].

Electrocatalytic hydrogen production has gradually emerged as a research hotspot due to its efficiency, controllability, and scalability. The electrocatalytic hydrogen production technology utilizes electrical energy to drive the electrolysis reaction of water molecules on the surface of a catalyst, decomposing water into hydrogen and oxygen. This process not only produces no pollutants but can also be powered by electricity generated from renewable energy sources such as solar and wind energy, thus achieving clean energy conversion and storage [16]. Similarly, efficient catalysts are also essential for electrocatalysis. Although noble metals and their composites are costly, they exhibit excellent catalytic performance and durability, making them crucial for electrocatalytic water splitting [17]. However, research efforts are ongoing to minimize the reliance on precious metals or find viable alternatives. Non-noble metal catalysts, such as transition metals and their compounds, offer a cost-effective and abundant solution, but their catalytic activity and stability still require further enhancement [18]. Novel catalysts have emerged as promising alternatives due to their unique properties and potential for high-performance electrocatalysis. In particular, single-atom catalysts have garnered significant attention for their exceptional catalytic performance and the potential to overcome the limitations of noble metal catalysts [19]. Perovskite materials, with their tunable composition and structure, exhibit great potential in electrocatalytic water splitting as well as broader energy conversion and storage applications [20]. With continuous advancements in materials science and engineering technology, the performance of these catalysts is expected to be further optimized, significantly improving the efficiency and economics of electrocatalytic hydrogen production, thus paving the way for large-scale industrial applications in green hydrogen production.

Despite the significant progress made, numerous challenges still exist in the field of photo(electro)catalytic water splitting, such as the activity, stability, cost of catalysts, and the feasibility of large-scale applications [6]. These issues limit the efficiency and economics of green hydrogen production, hindering its widespread commercialization. Therefore, this paper summarizes some of the latest research achievements in photo(electro)catalytic water splitting, offering a comprehensive perspective on the scientific principles and engineering applications of this technology. Particularly, it focuses on the design and optimization of catalysts, as well as the economic and technical challenges that may be encountered in practical applications. This paper can provide reference on future research and the practice of hydrogen production technologies for scholars and industries.

## 2. Mechanism

### 2.1. Photocatalysis

Plotnikow first coined the term “photocatalysis” in 1910, and Landau’s research on photocatalytic phenomena in 1913 laid the foundation for the formation and development of the field of photocatalysis [21]. In 1972, with the application of TiO₂ in photocatalytic oxidation and water splitting, the field of hydrogen production through photocatalytic water splitting also began to develop rapidly [9].

#### 2.1.1. Mechanism of Photocatalytic Reaction

As illustrated in Figure 1D, the medium for photocatalytic water splitting is a semiconductor catalyst [8]. The reaction process on the photocatalyst can be divided into three steps: (1) firstly, upon light excitation, photogenerated electrons (e^−^) transition from the valence band (VB) to the conduction band (CB) of the semiconductor, leaving photogenerated holes (h^+^) in the valence band; (2) subsequently, the e^−^ and holes migrate to the photocatalyst surface; and (3) finally, the e^−^ and holes participate in the two half-reactions of H_2_O reduction to produce H_2_ (HER) and H_2_O oxidation to produce O_2_ (OER), respectively. The relevant chemical reactions are shown in the following equations [22].

HER: 2H^+^ + 2e^−^ → H_2_ (1)

OER: 2H_2_O + 4h^+^ → O_2_ + 4H^+^ (2)

Total equation: 2H_2_O → O_2_ + 2H_2_ (Δ*G*_r_ = 273 kJ·mol^−1^, *T* = 298 K) (3)

**Figure 2:**
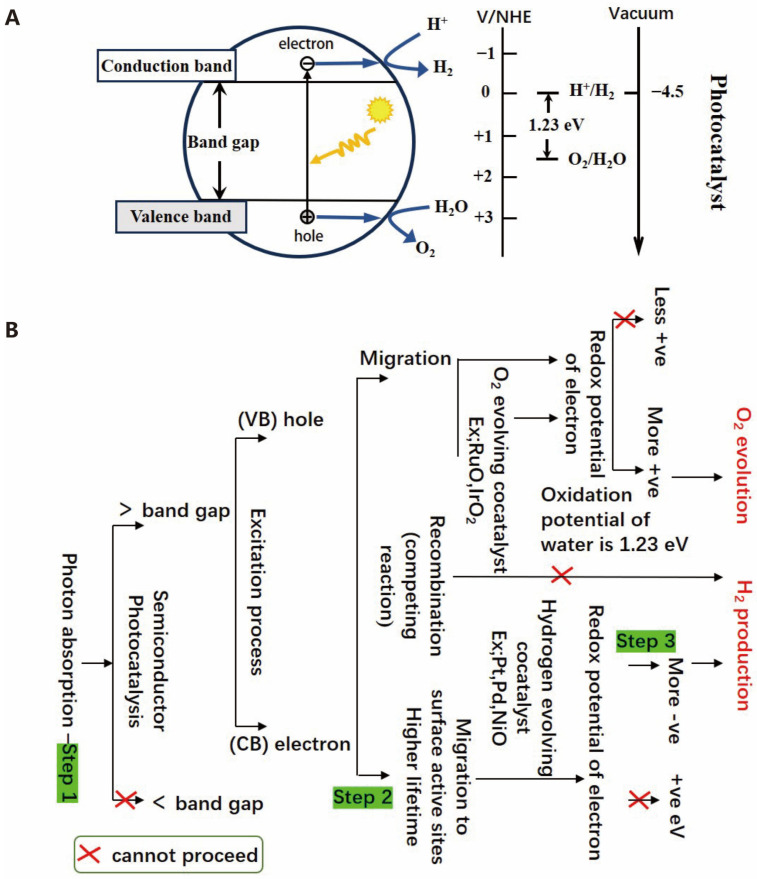
(**A**) Energy band of semiconductor-based photocatalytic water splitting for H_2_ and O_2_ evolution (adapted from [23] with permission from American Chemical Society, copyright 2010). (**B**) Flowchart representation of the basic and critical processes involved in heterogeneous semiconductor photocatalysis (adapted from [24] with permission from Springer Nature Link, copyright 2015).

Furthermore, the occurrence of photocatalytic reactions also requires the fulfillment of thermodynamic conditions. Specifically, the band structure of the photocatalyst must span the reaction potentials, and an overpotential is essential to ensure the occurrence of photocatalytic oxidation and reduction reactions. As shown in Figure 2A, the conduction band minimum (CBM) potential of the photocatalyst must be more negative than that of photoreduction reaction, and the valence band maximum (VBM) potential must be higher than that of the oxidation reaction [23]. For water splitting, the CBM potential of the photocatalyst should be more negative than the H^+^/H_2_ potential of 0 V for the electrons in the conduction band to reduce protons to hydrogen, and the VBM potential must be more positive than the O_2_/H_2_O potential of 1.23 V for the holes to oxidize water.

#### 2.1.2. Steps of Photocatalytic Reaction

**Figure 3:**
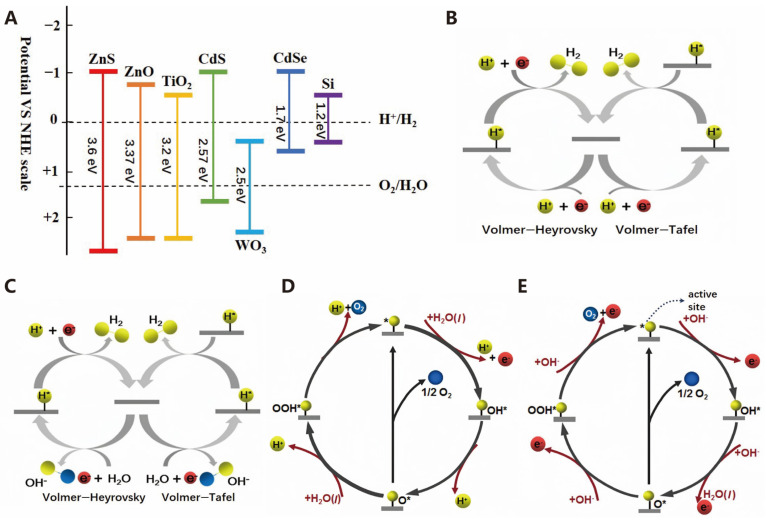
(**A**) Band gap potential diagram of semiconductors at normal hydrogen electrode (NHE) scale (adapted from [24] with permission from Springer Nature, copyright 2015). Schematic diagram of HER mechanism, (**B**) in acid electrolyte, and (**C**) in alkaline electrolyte. Schematic diagram of OER mechanism, (**D**) in acidic environment, and (**E**) in alkaline environment (reproduced from [17] with permission from Elsevier, copyright 2022).

As shown in Figure 2B, photocatalytic technology activates chemical reactions by utilizing light energy, and its process involves three core steps [24]. Firstly, in the light excitation stage, the photocatalyst absorbs light energy, prompts the electrons to transition from the valence band to the conduction one, and forms electron–hole pairs (various semiconductor band edge potentials at the NHE scale are shown in Figure 3A). Secondly, the electrons and holes separate on the catalyst surface in the charge separation stage. Lastly, the surface reaction stage, where the separated electrons and holes participate in redox reactions, generates active species that subsequently catalyze other chemical reactions [25,26]. These three steps work together, enabling photocatalytic technology to demonstrate broad application potential in various fields such as environmental purification, solar energy conversion, and organic synthesis.

### 2.2. Electrocatalysis

In 1789, Deiman and Van Troostwijk, using an electrostatic device to generate electricity, first achieved the water electrolysis on a gold electrode in a Leyden jar and observed the production of gas [27]. This was the early prototype of the water electrolysis, marking the beginning of the water decomposition exploration under the action of an electric current. Currently, the global share of hydrogen produced via water electrolysis stands at approximately 4% of the total hydrogen output worldwide [28]. With the development of hydrogen energy technology, its application prospects are becoming increasingly promising.

#### 2.2.1. Mechanism of Electrocatalytic Hydrogen Evolution Reaction

In the process of water electrolysis, the key half-reaction for hydrogen production at the cathode is the hydrogen evolution reaction (HER). During this process, a transfer of two electrons occurs, leading to the reduction in protons or water into hydrogen gas (H_2_) when an external voltage or electrochemical energy source is utilized. The mechanism is largely influenced by environmental factors [29]. Additionally, the hydrogen evolution process in water electrolysis consists of multiple steps, and the reaction pathways differ in acidic and alkaline environments. The HERs in acidic media and alkaline media are shown in Figure 3B and Figure 3C, respectively [17]. Generally, the HER in water electrolysis can be divided into two steps. The first step is the Volmer reaction, where H^+^ adsorbs onto the catalyst and binds to an active site to form H^*^. Then, the Heyrovsky reaction occurred in the second stage, where H^*^ combines with another H and an electron to form H_2_. Alternatively, in the Tafel reaction, two H^*^ on the catalyst surface combine to form H_2_ [30]. The reaction equations are as follows:

Volmer Reaction:

Acid solution: H^+^ + M + e^−^ ↔ M–H^*^ (4)

Alkaline solution: H_2_O + M + e^−^ ↔ M–H^*^ + OH^−^ (5)

Heyrovsky Reaction:

Acid solution: M–H^*^ + H^+^ + e^−^ ↔ H_2_ + M (6)

Alkaline solution: M–H^*^ + H_2_O + e^−^ ↔ M + OH^−^ + H_2_ (7)

Tafel Reaction:

Acid or Alkaline solution: 2M–H^*^ ↔ 2M + H_2_ (8)

where M is the surface-active site, and H^*^ represents the hydrogen atom adsorbed onto M.

Research has found that the selectivity of the desorption process is related to the chemical properties and surface electronic properties of the catalyst material [30]. It is reported that HER activity is associated with hydrogen adsorption, whose free energy is considered a key parameter for hydrogen evolution materials, and an optimal hydrogen binding energy facilitates the HER process [31].

#### 2.2.2. Mechanism of Electrocatalytic Oxygen Evolution Reaction

The oxygen evolution reaction (OER) entails the transfer of four e^−^, involving the O-H bond break and O-O bond formation. As such, the OER is a thermodynamically challenging process due to the needs for a substantial potential to surmount kinetic barriers [32]. Currently, the OER remains a bottleneck in water electrolysis, and its reaction mechanism can vary depending on the electrolyte pH [33]. Figure 3D represents the OER in acidic media, while Figure 3E represents the OER in alkaline media [17].

Acid solution:

2H_2_O → O_2_ + 4H^+^ + 4e^−^ (9)

H_2_O + ^*^ → OH^*^ + H^+^ + e^−^ (10)

OH^*^ → O^*^ + H^+^ + e^−^ (11)

2O^*^ → 2^*^ + O_2_ (12)

O^*^ + H_2_O → OOH^*^ + H^+^ + e^−^ (13)

OOH^*^ → ^*^ + O_2_ + H^+^ + e^−^ (14)

Alkaline solution:

4OH^−^ → 2H_2_O + O_2_ + 4e^−^ (15)

OH^−^ + ^*^ → OH^*^ + e^−^ (16)

OH^*^ + OH^−^ → O^*^ + H_2_O + e^−^ (17)

2O^*^ → 2^*^ + O_2_ (18)

O^*^ + OH^−^ → OOH^*^ + e^−^ (19)

OOH^*^ + OH^−^ → ^*^ + O_2_ + H_2_O + e^−^ (20)

In both acidic and alkaline solutions, the OER process produces the same intermediates (O^*^and OOH^*^), but the way these intermediates ultimately form oxygen differs [34]. The reaction equations are as shown above, where (*) is the active site.

## 3. Classification of Photo(electro)catalytic Materials

### 3.1. Photocatalysis

#### 3.1.1. Oxides

**Figure 4:**
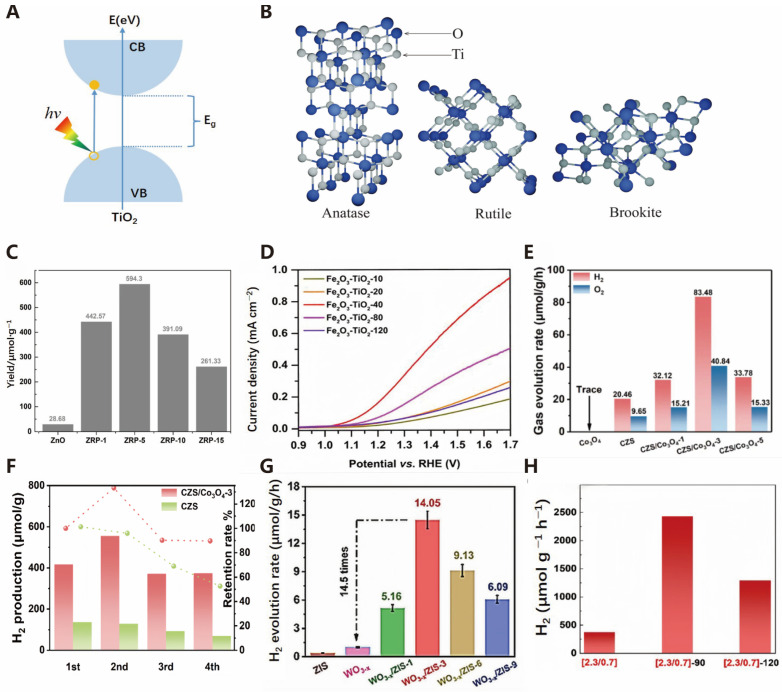
(**A**) Schematic diagram of the photoexcitation of electrons during TiO_2_ photocatalysis (adapted from [44] with permission from John Wiley & Sons, copyright 2021). (**B**) Crystal structure of TiO_2_ polymorphs—anatase, rutile, and brookite [44]. (**C**) Photocatalytic H_2_ release yield in 6 h [42]. (**D**) J-V curves of Fe_2_O_3_-TiO_2_-10, Fe_2_O_3_-TiO_2_-20, Fe_2_O_3_-TiO_2_-40, Fe_2_O_3_-TiO_2_-80, and Fe_2_O_3_-TiO_2_-120 (reproduced from [43] with permission from John Wiley & Sons, copyright 2018). (**E**) Photocatalytic overall water splitting of Co_3_O_4_, CZS, and CZS/Co_3_O_4−x_. (**F**) Cyclic stability experiments of CZS and CZS/Co_3_O_4_-3 (reproduced from [45] with permission from Elsevier, copyright 2023). (**G**) Time-dependent photocatalytic H_2_ evolution rates of ZIS, WO_3−x_, and WO_3−x_/ZIS composites under NIR (λ > 800 nm) light irradiation (reproduced from [46] with permission from Elsevier, copyright 2024). (**H**) H_2_ production by (2.3/0.7) ZrO_2−x_ catalyst under various oxidation times (400 °C) [47].

TiO_2_ is an excellent semiconductor material that boasts benefits like strong redox capacity, superior chemical reactivity, exceptional efficiency, chemical stability, harmlessness, straightforward synthesis process, and cost-effectiveness. It is widely used in photocatalytic reactions [35]. The light absorption properties of TiO_2_ are determined by its band gap value, while its redox capability is jointly determined by the band edge positions of the conduction band (CB) and valence band (VB). Figure 4A shows electrons being excited by light absorption. Because TiO_2_ generates charge carriers (electrons and holes) upon UV absorption, owing to its band gap and high refractive index, it is a promising material for applications like solar water splitting, photocatalyzed decomposition, hydrogen storage, sensors [36], and bactericides [37]. The TiO_2_ crystal structure primarily comprises anatase, brookite, and rutile phases (Figure 4B). Studies have found that anatase has higher photocatalytic efficiency compared to rutile under ultraviolet light irradiation. The primary crystal faces observed in the anatase structure are (011) and (001), with the (001) face demonstrating significant reactivity and influencing the catalyst’s activity, stability, and adsorption properties [36]. However, the high band gap width and fast photogenerated carrier recombination rate of TiO_2_ itself significantly affect the catalyst’s hydrogen production efficiency.

Researchers often employ three methods to improve this catalyst: surface structure modification, metal/non-metal ion doping, and precious metal deposition [38]. In recent years, the study of Fe^3+^ doping in titanium dioxide has received widespread attention in enhancing the photocatalytic efficiency of TiO_2_. According to research conducted by Litter et al. [39], it is generally believed that the mechanism for this improvement is due to Fe^3+^ ions replacing Ti^4+^ ions, forming electron and hole trap sites in the TiO_2_ matrix and on the particle surface. This helps reduce the electron–hole recombination and extends absorption into the visible light spectrum. Additionally, combining TiO_2_ with suitable semiconductors through heterojunction methods can enhance photocatalytic efficiency [40]. Table 1 presents the hydrogen production efficiencies of various TiO_2_ composite photocatalysts [41].

In addition to titanium dioxide, other oxides such as ZnO, Fe_2_O_3_, Co_3_O_4_, WO_3_, ZrO_2_, etc., have also made significant progress in photocatalysis research. However, metal oxide bulks possess notable band gaps that limit their ability to absorb visible light. To address this issue, researchers have adopted different strategies to improve the visible light responsiveness and photocatalytic efficiency of these materials. ZnO, with its inherently wide band gap, can only absorb the ultraviolet region of the solar spectrum (about 4%), resulting in low photoelectric conversion efficiency during the initial light absorption stage of the photocatalytic process. Furthermore, ZnO is prone to photocorrosion during photocatalysis, affecting its stability and reusability. Chen et al. [42] prepared a ZnO/RP heterostructure through a simple thermal treatment method and investigated how varying the ratio of red phosphorus affected the photocatalytic activity. The ZnO/RP heterostructure demonstrated a significant improvement in hydrogen production, reaching up to 20.8 times that of pure ZnO (Figure 4C). The improved photocatalytic performance is primarily due to the efficient migration of photogenerated electron–hole pairs and the inhibition of surface charge carrier recombination. Additionally, the ZnO/RP heterostructure exhibited good photostability during the photocatalytic reaction, overcoming the photocorrosion issue of ZnO. In Fe_2_O_3_, photogenerated electrons and holes are prone to recombination, resulting in low charge separation efficiency and affecting its photocatalytic performance. During the photocatalytic process, photocorrosion may occur, where the catalyst itself undergoes chemical changes under illumination and water oxidation conditions, resulting in the degradation of its structure and performance. Zang et al. created Fe_2_O_3_-TiO_2_ microdumbbells by growing amorphous TiO_2_ nanospheres on both ends of Fe-based metal–organic compound (FeMOC) microrods using a simple synthesis method involving hexadecylamine molecules [43]. As shown in Figure 4D, the compositionally and structurally optimized Fe_2_O_3_-TiO_2_-40 micrometer dumbbells exhibit the highest photocurrent density. Furthermore, the combination of Fe_2_O_3_ and TiO_2_ enables the response to light of different wavelengths, thereby enhancing light absorption capability.

In Co_3_O_4_ material, photogenerated electrons and holes readily recombine, which limits the enhancement of its photocatalytic performance. Yu et al. successfully loaded Co_3_O_4_ nanoparticles onto one-dimensional Cd0.6Zn0.4S nanorods using a simple hydrothermal method, preparing a CZS/Co_3_O_4_ composite photocatalyst that achieves efficient water splitting [45]. The Co_3_O_4_ nanoparticles can effectively capture photogenerated holes, effectively inhibiting photocorrosion of the CZS nanorods, promoting rapid surface charge transfer, and significantly extending the lifetime of photogenerated carriers. Experimental results demonstrate that the CZS/Co_3_O_4_ photocatalyst exhibits high hydrogen and oxygen production rates of 83.48 μmol g^−1^h^−1^ and 40.48 μmol g^−1^h^−1^, respectively, in pure water (Figure 4E), and shows good stability over four consecutive reaction cycles (Figure 4F). WO_3_ has a band gap of approximately 2.6–2.8 eV, which means it primarily absorbs ultraviolet light and has a weaker ability to absorb visible light. Due to the low proportion of ultraviolet light in sunlight, this limits the efficiency of WO_3_ in converting solar energy into chemical energy. Chen et al. [46] evaluated the photocatalytic hydrogen production efficiency of WO_3−x_, ZIS, and WO_3−x_/ZIS composites under near-infrared light irradiation. The study found that the WO_3−x_/ZIS-3 composite exhibited the best photocatalytic activity under near-infrared light, with a hydrogen generation efficiency of 14.05 μmol g^−1^h^−1^ (Figure 4G). The surface of zirconia (ZrO_2_) may lack sufficient active sites for adsorbing and activating water molecules, which is a prerequisite for water splitting reactions. Asterios et al. [47] activated nanoscale ZrO_2_-x with engineered lattice vacancies (Vo) to absorb solar light, achieving a hydrogen (H_2_) production rate exceeding 2400 μmolg^−1^h^−1^ (Figure 4H).

#### 3.1.2. Sulfides

**Figure 5:**
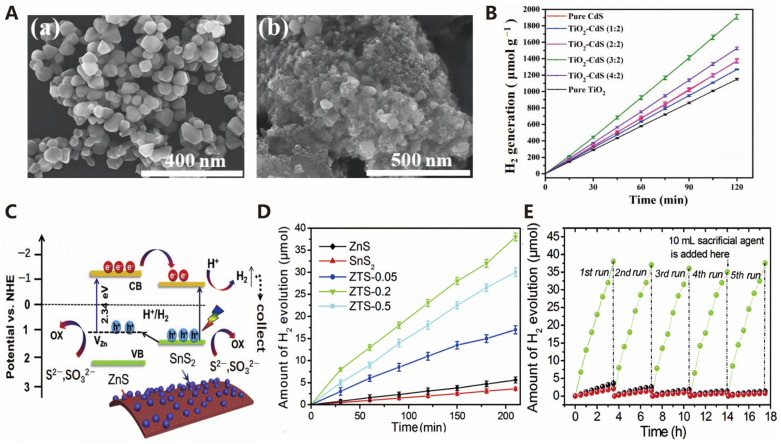
(**A**) FE-SEM images of (**a**) pure CdS nanoparticles, (**b**) TiO_2_-CdS (3:2), and (**B**) the photocatalytic H_2_ generation rate by using pure CdS nanoparticles, pure TiO_2_ and TiO_2_-CdS samples under simulated solar light at AM 1.5G conditions (reproduced from [51] with permission from Elsevier, copyright 2019). (**C**) The band gap structure and photocatalytic H_2_ production mechanism of the porous ZTSx nanosheet catalyst under visible light irradiation. (**D**) The photocatalytic hydrogen evolution amount of SnS_2_ and ZTSx porous nanosheets in 210 min with 20 mg photocatalysts. (**E**) The amount of H_2_ evolution by ZnS (black), SnS_2_ (red), and ZTS-0.2 nanosheets (green) for five photocatalyst experiment cycles (reproduced from [53] with permission from Elsevier, copyright 2018).

Metal sulfides have garnered widespread attention as catalysts for photocatalytic water splitting to produce hydrogen due to their unique physicochemical properties. These materials typically possess suitable band structures, excellent visible light response, and tunable photoelectrochemical properties, making them exhibit good catalytic activity in photocatalytic water splitting for hydrogen production [48]. For many years, CdS and ZnS metal sulfide catalysts have been the focus of considerable attention [49]. CdS is a promising photocatalyst for water splitting that responds to visible light, owing to its smaller band gap of 2.4 eV compared to metal oxide semiconductors [50]. In addition, Yang et al. synthesized a TiO_2_-CdS nanocomposite with a core–shell structure (Figure 5A). The hydrogen production rate reached 954 µmol g^−1^h^−1^, which is approximately 1.4 times and 1.7 times higher than that of pure CdS nanoparticles and pure TiO_2_, respectively (Figure 5B) [51]. ZnS is also an important photocatalytic material that has been extensively studied as a photocatalyst due to its photoelectric effect in the ultraviolet to visible light region [52]. In terms of photocatalytic water splitting for hydrogen production, ZnS exhibits chemical stability and a suitable band structure, but strategies such as doping, introducing defects, and constructing heterojunctions are needed to improve its photocatalytic efficiency and stability. Wang et al. synthesized two-dimensional (2D) ZnS-SnS_2_ porous nanosheets as photocatalysts and elucidated the photocatalytic hydrogen production mechanism of this catalyst (Figure 5C). The nanosheets have a high specific surface area of 246.7 m^2^/g and a photocatalytic hydrogen production rate of 536 mmol g^−1^h^−1^, which are 10 times and 17 times those of ZnS and SnS_2_, respectively (Figure 5D). Furthermore, the catalyst exhibits good stability, with its activity and structure remaining stable and showing no significant changes after at least five cycles of use (Figure 5E) [53].

#### 3.1.3. Nitrides

Nitrides and nitrogen oxides, as photocatalysts, can effectively harvest solar energy for water splitting [54]. Nitrides, with their higher 2p orbital energy of nitrogen, easily excite electrons into the conduction band; however, they exhibit poor stability in water and are not suitable for direct use in water splitting [55]. Metal nitrogen oxides, combining the advantages of a low band gap and high stability in water, have been developed as effective visible-light-driven photocatalysts for water splitting.

**Figure 6:**
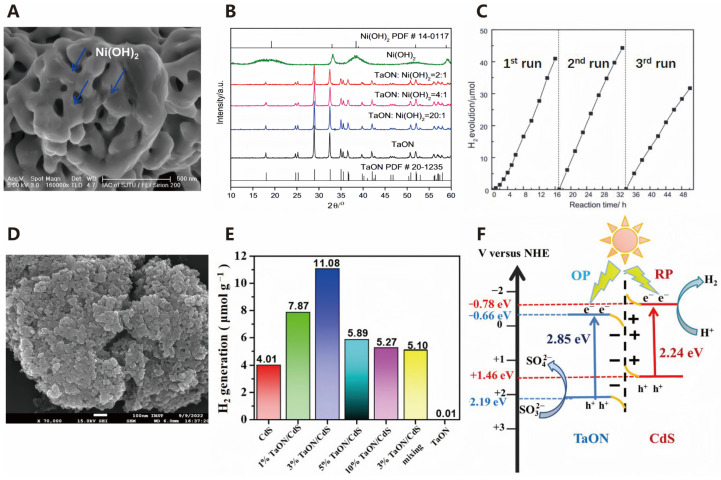
(**A**) SEM images of Ni(OH)_2_/TaON. (**B**) XRD patterns of TaON, Ni(OH)_2_, and Ni(OH)_2_/TaON composite materials with different weight ratios of Ni(OH)_2_. (**C**) Time courses of photocatalytic hydrogen evolution on Ni(OH)_2_/TaON (TaON:Ni(OH)_2_ = 5) under visible light (λ > 400 nm) (reproduced from [59] with permission from Elsevier, copyright 2015). (**D**) FESEM images of 3% TaON/CdS. (**E**) Hydrogen evolution rate of CdS, TaON/CdS composite, and TaON nanomaterials. (**F**) Schematic illustration of photocatalytic hydrogen evolution S-scheme mechanism over TaON/CdS nanocomposites (reproduced from [60] with permission from Elsevier, copyright 2023).

Over the past 40 years, more than 130 semiconductor materials have been reported as efficient photocatalysts for hydrogen production through water splitting, but most are only active under ultraviolet light. In recent years, non-oxide materials such as sulfides and nitrogen oxides have been found to be active for producing H_2_ and O_2_ under visible light. However, the quantum efficiency of visible-light-driven photocatalysts remains low, which is a bottleneck for solar hydrogen production [56]. Therefore, the research focus is on developing visible-light-responsive and stable photocatalysts for water splitting [57]. Transition metal nitrogen oxides with d^0^ and d^10^ electronic configurations are potential candidates, as their valence bands are composed of hybridized N_2p_ and O_2p_ orbitals, and their conduction bands are primarily composed of empty d orbitals of the metal ions, resulting in more negative valence band energy levels and smaller band gaps [58]. Chen et al. loaded Ni(OH)_2_ onto TaON via a precipitation method to enhance its photocatalytic water splitting activity under visible light irradiation. Subsequently, they characterized the prepared photocatalyst using techniques such as scanning electron microscopy (SEM, Figure 6A) and X-ray diffraction (XRD, Figure 6B). The experimental results showed that the hydrogen production rate of this photocatalyst was twice that of 0.5% Pt/TaON, reaching 3.15 µmol/h (Figure 6C) [59]. In addition, Kalia et al. synthesized TaON/CdS heterojunction photocatalysts via a precipitation method (Figure 6D). The 3% TaON/CdS heterostructure exhibited the highest hydrogen production activity under visible light irradiation, reaching 11.08 mmol/h (Figure 6E). S-type heterojunctions were formed between the nanocomposites, effectively promoting the separation of electron–hole pairs and thereby enhancing the photocatalytic hydrogen production efficiency (Figure 6F) [60].

**Figure 7:**
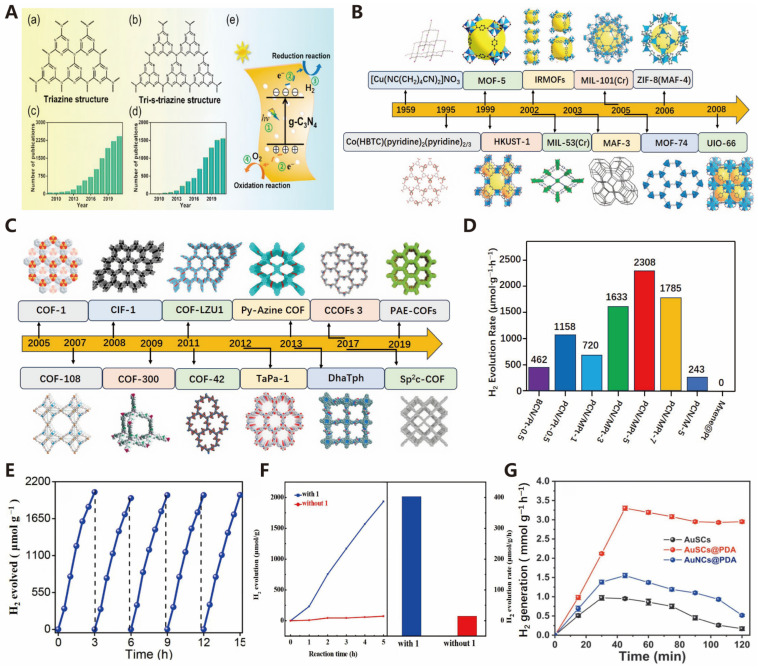
(**A**) (**a**) Triazine structure and (**b**) Tris-s-triazine structure of g-C_3_N_4_. (**c**) The number of articles published on the topic of “graphitic carbon nitride” and (**d**) The number of articles published on the topic of “graphitic carbon nitride + photocatalytic” from 2009 to 2021. (**e**) Schematic diagram illustrating principle of photocatalytic process of g-C_3_N_4_ based photocatalysts [66]. (**B**) The classic materials in the history of MOF development (adapted form [13] with permission). (**C**) The classic materials in the history of COF development (adapted from [13] with permission). (**D**) The photocatalytic H_2_ evolution activity of BCN/Pt-0.5, PCN/Pt-0.5, PCN/MPt-x, and PCN/M-5 samples with methanol as sacrificial agent (adapted from [83] with permission from Elsevier, copyright 2023). (**E**) Five hydrogen production cycles using the composite PCN-20 (reproduced from [84] with permission from Elsevier, copyright 2021). (**F**) The comparison of H_2_ evolution profiles under UV–visible (UV–vis) light, obtained with or without 1 (reproduced from [88] with permission from Elsevier, copyright 2021). (**G**) Time-dependent photocatalytic H_2_ evolution for AuSCs@PDA (reproduced from [89] with permission from John Wiley & Sons, copyright 2024).

Graphitic carbon nitride (g-C_3_N_4_) has attracted widespread attention due to its excellent physicochemical properties [61]. As shown in Figure 7A, this material exhibits a two-dimensional layered structure similar to graphene, consisting of π-conjugated planes formed by sp^2^-hybridized C and N atoms [62]. Its basic structural units are triazine (C_3_N_3_) and tri-s-triazine (C_6_N_7_) [63]. g-C_3_N_4_ has a band gap of approximately 2.7 eV and suitable positions of conduction and valence bands, enabling it to respond to visible light. Since it is composed of abundant non-metallic elements on Earth, g-C_3_N_4_ is cost-effective for large-scale applications. It is widely used in various photocatalytic reactions, which typically involve three steps: light excitation to generate electron–hole pairs, the separation and migration of charge carriers to the surface, and redox reactions involving surface electrons and holes [64]. Nevertheless, the photocatalytic efficiency of g-C_3_N_4_ is limited by several factors, such as a high electron–hole recombination rate, the limited range of visible light absorption, few reactive sites, grain boundary effects, and low charge mobility [65]. To enhance its efficiency, researchers are working on increasing the number of photogenerated electron–hole pairs, enhancing charge separation and migration efficiency, and creating more active sites on the surface of the photocatalyst [66].

#### 3.1.4. Novel Photocatalyst

Metal–Organic Frameworks (MOFs) are inorganic–organic hybrid porous materials composed of metal nodes (metal ions or clusters) and organic linkers [67,68]. MOFs exhibit characteristics such as large internal surface areas, ultra-low densities, uniform cavity structures, and molecular-sized pores. As shown in Figure 7B, the origin of MOF materials can be traced back to the synthesis of the coordination network polymer [Cu(NC(CH_2_)_4_CN)_2_]NO_3_ by Yoshihiko et al. in 1959 [69]. These synthesized Metal–Organic Frameworks (MOFs) demonstrate excellent light absorption and energy transfer capabilities. These properties are crucial for sunlight capture and charge separation in solar energy conversion processes, making MOFs efficient photosensitizers in photocatalytic reactions and widely used in photocatalytic water splitting, CO_2_ reduction, and other related reactions [68]. Recent studies have shown that some functionalized MOFs serve as effective photocatalysts for visible-light-driven hydrogen production, with turnover numbers (TONs) reaching up to 7000 [70]. Furthermore, Toyao et al. showed that a Ti-based MOF doped with a Ru complex can be used for producing hydrogen under visible light illumination at a wavelength of 620 nm [71]. Moreover, incorporating functional amino groups into the structure of the Ti-based MOF led to a red shift in the visible light absorption band to approximately 500 nm, resulting in a substantial increase in catalytic activity [72].

Covalent Organic Frameworks (COFs) are composed of light elements such as carbon (C), hydrogen (H), oxygen (O), nitrogen (N), boron (B), etc., connected through relatively high-energy covalent bonds [73], exhibiting advantages such as low density, high porosity, and high thermal and chemical stability [74]. As shown in Figure 7C, in 2005, Yaghi et al. first reported COF materials and synthesized COF-1 and COF-5 with specific topological structures [75]. Compared to Metal–Organic Frameworks (MOFs), COF elements demonstrate distinct asymmetry and correlation in photocatalytic HER. Additionally, the covalent bonds in the COF framework significantly enhance their durability. However, converting highly covalently linked COFs into large-scale crystalline materials remains a challenge [76]. Utilizing solar energy to promote water splitting into H2 and O2 is widely regarded as a promising technology for producing environmentally friendly H2. To achieve this, catalysts with appropriately arranged bonds are needed to effectively convert photons into chemical energy, providing sufficient reaction possibilities [77]. Covalent Organic Frameworks (COFs), due to their unique properties, can meet the demand for efficient charge separation [78]. The key to improving photocatalytic efficiency lies in the effective separation and transfer of photogenerated charges. Currently, the solar-to-hydrogen (STH) efficiency of COF photocatalysts is far from the theoretical value [79]. Many pure COF photocatalysts exhibit relatively low photocatalytic efficiencies, while higher-efficiency COF-based photocatalysts are often prepared by combining COFs with other materials (such as Metal–Organic Frameworks (MOFs), MXenes, etc.), although this composite process increases the difficulty of photocatalyst synthesis [80].

MXenes are a type of two-dimensional inorganic materials consisting of transition metal carbides, nitrides, or carbonitrides, characterized by their unique structural flexibility, diverse elemental composition, excellent electrical conductivity, outstanding carrier mobility, and abundant catalytic active sites. These properties enable MXenes to accelerate interfacial charge transfer and inhibit photogenerated charge recombination in photocatalytic reactions, thereby enhancing photocatalytic efficiency [81,82]. In recent years, notable advancements have been achieved in the study of MXenes within the photocatalysis domain. Researchers have developed various efficient MXene-based photocatalysts through different synthesis methods and strategies. For instance, MXenes can be utilized as non-precious metal co-catalysts in photocatalytic reactions, providing a viable substitute for expensive precious metal catalysts. Huang et al. found that the PCN samples modified with MXene@Pt (PCN/MPt-x) exhibited good hydrogen production activity, and this activity was closely related to the content of the MXene@Pt co-catalyst. As shown in Figure 7D, the sample with the highest activity was PCN/MPt-5, with a hydrogen production rate of up to 2308 μmol g_cat_^−1^h^−1^, exceeding that of PCN modified with an equivalent amount of Pt and PCN modified with pristine MXene. This indicates that the MXene@Pt co-catalyst provides excellent light harvesting capability, the rapid capture of photogenerated charges, and abundant active sites [83]. Furthermore, MXene can also form composite photocatalytic systems with other materials such as TiO_2_, CdS, and g-C_3_N_4_ by constructing heterojunction structures, which expand the spectrum of light absorption and boost photocatalytic efficiency. Xu et al. developed a novel PCN/Ti_3_C_2_ MXene heterojunction that exhibited significantly enhanced hydrogen production rates in photocatalytic hydrogen generation. As illustrated in Figure 7E, under 420 nm illumination for 3 hours, the PCN-20 sample demonstrated a hydrogen production rate of approximately 2181 μmol g^−1^h^−1^ with a quantum efficiency of 8.6%, markedly higher than that of CN or unmodified PCN. Furthermore, PCN-20 maintained a stable hydrogen production rate over five cycles, attesting to the excellent stability of the heterojunction [84].

Nanoclusters are ultrafine particles at the nanoscale, representing relatively stable, non-rigid aggregates composed of a few to several hundred atoms or molecules. These nanoparticles typically have sizes ranging from 1 to 100 nanometers, bridging the gap between microscopic atoms/molecules and macroscopic solid materials. Nanoclusters can be categorized into metal nanoclusters, non-metal nanoclusters, and others. Among them, metal nanoclusters constitute an important branch, consisting of a few to dozens of metal atoms (such as Au, Ag, Cu, Pd, Pt, Ni, etc.) and exhibiting unique physical and chemical properties under the protection of organic ligands [85,86,87]. Due to their unique quantum size effects, surface geometric effects, and electronic properties, nanoclusters often exhibit satisfactory HER activity. These attributes allow nanoclusters to efficiently facilitate the separation and transport of photogenerated electron–hole pairs during both photocatalytic and electrocatalytic water splitting processes for hydrogen production, thereby enhancing catalytic efficiency. Recently, extensive research has been conducted on nanoclusters in the area of hydrogen production through photocatalytic water splitting. The size and shape of nanoclusters have a substantial influence on their photocatalytic efficiency. Smaller sizes and specific shapes can enhance the number of active sites on the cluster surfaces and improve light absorption efficiency, thereby enhancing photocatalytic activity. Liu et al. successfully synthesized a core–shell nanoring cluster [Al_7_Ti_14_(μ_2_-O)_7_(μ_3_-O)_14_(L)_35_] 2CH_3_CN (denoted as 1; L = benzoic acid) through the hydrolysis reaction of Ti and Al ions. This nanocluster possesses a rare odd-numbered ring structure and is currently the highest nuclearity aluminum-containing PTC known. As shown in Figure 7F, this compound demonstrated excellent photocatalytic activity in hydrogen production tests, with a hydrogen production rate of 402.88 μmol g^−1^h^−1^, representing the highest reported among PTC materials to date [88]. Furthermore, considerable research has been conducted on metal nanoclusters, including those made of Au, Ag, Pt, among others, with metal clusters serving as cocatalysts to enhance the separation efficiency of photogenerated charges. Bera et al. investigated an efficient photocatalytic hydrogen production method that utilizes assemblies of in situ polymerized gold nanoclusters (AuNCs) and gold superclusters (AuSCs). The research team successfully embedded AuNCs uniformly into a polydopamine (PDA) matrix by the in situ polymerization of dopamine (DA) in an alkaline aqueous medium, forming two distinct structures: AuNCs@PDA and AuSCs@PDA. These structures not only exhibited enhanced metallic properties but, also, after photopolymerization, the PDA layer acted as an efficient electron transport medium between adjacent AuNCs, reducing the recombination of excited state electrons. Specifically, they found that the AuSCs@PDA structure, compared to isolated AuSCs or PDA nanoparticles, demonstrated a larger potential difference (26.0 mV vs. 18.4 mV and 14.6 mV), indicating the accumulation of more photogenerated carriers. As a result, AuSCs@PDA achieved a higher photocurrent density, improved photostability, and lower charge transfer resistance. As shown in Figure 7G, the hydrogen generation rate reached 3.20 mmol g^−1^h^−1^ [89].

### 3.2. Electrocatalysis

#### 3.2.1. Noble Metals and Their Composites

Water electrolysis for hydrogen production is a technology that converts electrical energy into chemical energy to generate hydrogen. Noble metals such as platinum (Pt), ruthenium (Ru), and iridium (Ir) are widely used in the electrocatalytic water splitting process, particularly in the HER at the cathode and the OER at the anode, due to their excellent catalytic performance and durability. In proton exchange membrane (PEM) water electrolysis technology, these noble metal catalysts can meet the high requirements for activity and stability in the electrolytic cell [90]. A promising approach to boost HER activity of platinum group metal-based catalysts involves enhancing the intrinsic activity of individual catalytic sites. Lv et al. successfully fabricated ultra-thin, curved nanosheets of a Pd-Ir alloy with a thickness of approximately five atomic layers, resulting in exceptional electrocatalytic efficiency for hydrogen production [91]. The significant cost associated with these precious metal catalysts poses a major barrier to their widespread adoption. Consequently, future research efforts should focus on minimizing the reliance on precious metals or investigating viable alternative catalysts to substitute them.

**Figure 8:**
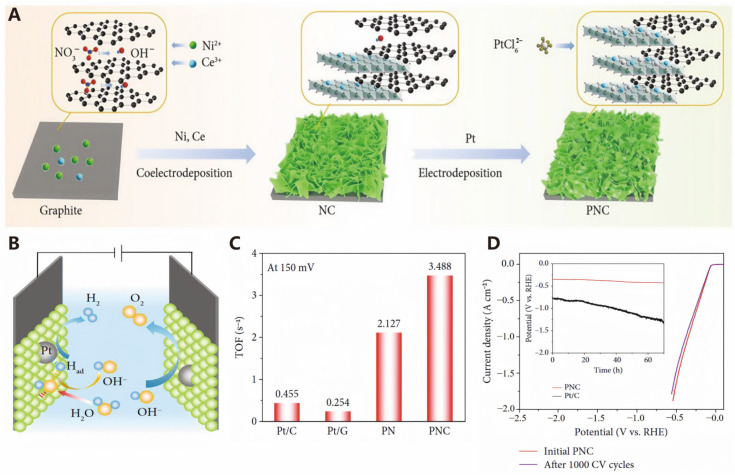
(**A**) Preparation diagram of PNC electrocatalyst on graphite. (**B**) Illustration of the PNC as a bifunctional electrode for overall water electrolysis. (**C**) Turnover frequency (TOF) deduced from the LSV curves at overpotential of 150 mV. (**D**) LSV curves of PNC before and after 1000 cycles [92].

Among the different metal hydroxides, Ni(OH)_2_ distinguishes itself by facilitating water dissociation and enhancing hydrogen production. Liu et al. found that combining Ni(OH)_2_ with Pt produces a significant synergistic effect, leading to the development of a novel hybrid electrocatalyst—Pt-decorated Ni(OH)_2_/CeO_2_ (Figure 8A). Furthermore, these embedded nanosheets not only effectively catalyze the OER, but also enhance the HER activity of Pt through electronic modulation, further accelerating water splitting (Figure 8B). The turnover frequency (TOF) of this hybrid material is as high as 3.488 s^−1^ (Figure 8C). Additionally, this PNC hybrid material exhibits excellent performance at high current densities, with rapid bubble formation and release, and no catalyst degradation observed (Figure 8D), demonstrating its great potential for industrial applications [92].

#### 3.2.2. Non-Noble Metal Catalyst

Electrocatalytic water splitting is an important clean energy production technology, and research on non-noble metal catalysts holds great significance for reducing production costs and achieving large-scale applications. Non-noble metal catalysts primarily include transition metals and their compounds, such as oxides, hydroxides, sulfides, and phosphides of elements like manganese (Mn), nickel (Ni), and iron (Fe), etc. [18]. These catalysts are characterized by low cost, abundant resources, and environmental friendliness. However, compared to precious metal catalysts, their catalytic performance and durability still require enhancement.

Manganese-based composites, characterized by structural diversity and abundant active sites, exhibit excellent catalytic performance and have attracted widespread attention in academia [18]. Additionally, manganese ions exhibit multiple valence states that can be interconverted, which facilitates the formation of defects and enhances catalytic performance. In the research on manganese-based catalysts, design strategies employing manganese single atoms and manganese alloys are commonly adopted to boost catalytic activity. Single atoms can fully exploit active sites, thereby increasing the catalytic efficiency per unit [93]. The synergistic effects in manganese alloys can modify the electronic structure characteristics and optimize the binding energy of intermediates, thus enhancing the catalytic performance [94].

Non-noble metal catalysts show significant potential for use in electrocatalytic water splitting, yet they encounter several hurdles. Firstly, there is a need to further enhance their catalytic activity and stability to meet the demands of large-scale applications. Secondly, efforts must be made to reduce the production costs of the catalysts and improve their economic viability. Furthermore, research on the recovery and reuse of catalysts should be strengthened to minimize environmental pollution.

#### 3.2.3. Novel Electrocatalyst

The novel catalysts introduced above, including MOFs (Metal–Organic Frameworks), COFs (Covalent Organic Frameworks), nanoclusters, and MXenes, not only exhibit excellent performance in the field of photocatalytic water splitting but also have been extensively studied in the realm of electrocatalysis. MOFs, characterized by their high specific surface area, tunable pore structure, high crystallinity, and customizable functionalization, are widely researched for electrocatalysis. They can serve as carriers, introduce active metals, construct two-dimensional conductive COFs, form COF-based hybrids, and obtain carbon materials through COF pyrolysis, among other design strategies, for a range of electrocatalytic processes, including the HER, the OER, and the Carbon Dioxide Reduction Reaction (CO_2_RR) [95,96]. Nanoclusters, particularly metal nanoclusters, possess high catalytic activity and selectivity due to the high degree of unsaturated surface atoms. Gold nanoclusters directly used for electrocatalytic hydrogen evolution demonstrate an increase in mass activity for hydrogen evolution as the number of gold atoms in the cluster decreases [97]. MXenes, owing to their excellent metallic conductivity, hydrophilicity, high specific surface area, and superior electrochemical properties, emerge as promising electrocatalytic materials. Recent advancements in MXene-based electrocatalytic materials include strategies such as coupling with active materials or heteroatom doping, constructing 3D MXene structures or introducing interlayer spacers, protecting the edges of MXenes, or converting the surface of MXenes into stable active substances, to enhance catalytic performance and stability [98]. Currently, in addition to these four types of innovative catalysts, single-atom catalysts and perovskite catalysts are also receiving intensive research attention in the area of photo(electro)catalytic water splitting for hydrogen generation.

**Figure 9:**
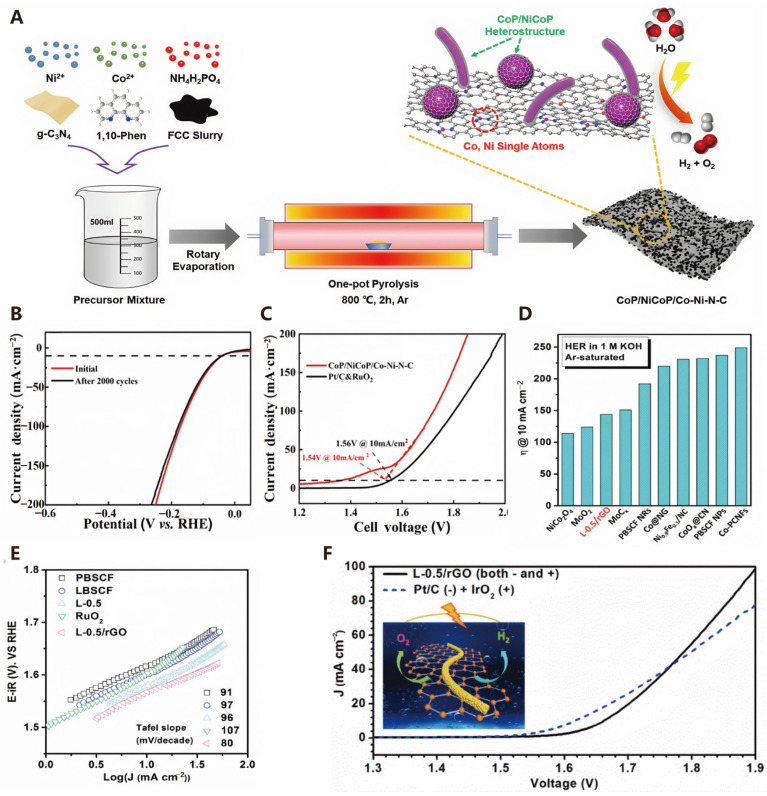
(**A**) Schematic illustration for the synthesis of CoP/NiCoP/Co-Ni-N-C. (**B**) Stability tests of CoP/NiCoP/Co-Ni-N-C toward the HER. (**C**) Polarization LSV curves of CoP/NiCoP/Co-Ni-N-C and noble-metal catalysts toward OWS (reproduced from [101] with permission from Elsevier, copyright 2024). (**D**) Comparisons of the IR-corrected HER activities of the L-0.5/rGO catalyst with other famous electrocatalysts in 1 m of KOH. (**E**) The IR-corrected Tafel plots of various catalysts in Ar-saturated 1 M KOH solutions. (**F**) The L-0.5/rGO nanohybrid as a bifunctional catalyst in an Ar-saturated 1 m KOH solution for overall water splitting (without IR-correction) (reproduced from [103] with permission from John Wiley & Sons, copyright 2017).

Single-atom catalysts (SACs) have garnered widespread attention due to their unique active centers and exceptional catalytic performance. These catalysts achieve a uniform dispersion of metal atoms on high-surface-area solid supports through coordination, embedding, adsorption, and other immobilization methods, exhibiting outstanding catalytic activity and selectivity. In photoelectrochemical (PEC) water splitting, metal catalysts that are atomically dispersed and anchored on photoelectrodes offer a significant advancement over conventional thin-film PEC catalysts, as they increase the number of catalytic sites and enhance the kinetics of photogenerated charge carriers [99]. SACs demonstrate excellent performance in electrocatalytic water splitting (HER and OER), and research progress indicates that they can achieve efficient catalysis through synergistic effects between single-atom sites and supports [100]. Liu et al. studied a bifunctional electrocatalyst (CoP/NiCoP/Co–Ni–N–C) prepared through a one-pot pyrolysis method. This catalyst integrates a CoP/NiCoP heterostructure with Co and Ni single atoms (Figure 9A). The electronic synergy between these components enables an efficient HER and overall water splitting while also demonstrating good stability (Figure 9B). There is a significant charge transfer between the heterostructure and the single atom (SA) sites, optimizing the charge distribution and achieving moderate H^*^ adsorption. As shown in Figure 9C, it exhibits a low overpotential of 40 mV at 10 mA cm^−2^ for the HER in 1 M of KOH. Furthermore, the two-electrode system requires only 1.54 V to reach a current density of 10 mA cm^−2^, significantly enhancing the efficiency of hydrogen production [101]. Typically, platinum-based catalysts are among the most efficient for the HER, owing to their high current density, low overpotential, and robust stability. Nevertheless, their widespread adoption is hindered by their high cost and limited availability. To tackle these challenges, numerous researchers have focused on developing HER electrocatalysts that utilize low loadings of precious metals. Notably, single-atom catalysts (SACs) emerge as a promising approach to overcome these limitations, as they have the potential to demonstrate outstanding HER activity [102].

Perovskite materials have the same crystal structure as calcium titanate (CaTiO_3_), named after the Russian mineralogist Lev Perovski. The term now refers to a series of materials with this crystal structure. The chemical composition of perovskite-type materials is abbreviated as ABX_3_, where the A, B, and X sites can be iteratively substituted, offering a wide range of material options that are both abundant and inexpensive. By adjusting the halogen content, the bandgap width of perovskites can be tuned, enabling them to absorb a broader spectrum of light, thus holding great potential for photocatalytic water splitting to produce hydrogen [104]. However, transition metal perovskite oxides (ABO_3_) are considered strong contenders for water electrolysis applications due to their abundance, oxidation resistance, and excellent catalytic efficiency. Specifically, the *e_g_* orbital electron filling of the B-site metal ions significantly influences the binding energy of intermediates, thereby enhancing electrocatalytic performance [105,106,107]. The perovskite structure exhibits flexibility in accommodating diverse dopants, and the electrocatalytic efficiency of perovskites is closely related to their characteristics, oxidation states, and the sites where doping occurs. Targeted and functional doping/replacement at the A-, B-, and/or O-sites of perovskite oxides constitutes an effective approach to attaining high-performance OER/HER water splitting [108]. Among the various perovskite oxides, Ba_0_._5_Sr_0_._5_Co_0_._8_Fe_0_._2_O_3−δ_ (BSCF) stands out as a particularly appealing compound, demonstrating exceptionally high activity for OERs/HERs, owing to its optimal filling of the *e_g_* orbitals [109]. For example, Hua et al. achieved excellent catalytic performance and stability by co-doping La/Ca into the A-site of BSCF to form the La_0.5_(Ba_0_._4_Sr_0_._4_Ca_0_._2_)_0_._5_Co_0_._8_Fe_0_._2_O_3−δ_ (L-0.5) perovskite oxide (Figure 9D,E). As shown in Figure 9F, the L-0.5/rGO electrolyzer exhibited a working voltage of 1.76 V at 50 mA cm^−2^, comparable to that of an IrO_2_/C coupled with Pt/C at the same current density [103]. This result indicates that this electrocatalyst can be scaled up for practical electrolysis applications, offering low cost and high efficiency. Furthermore, combining perovskite oxides with other materials to form heterojunctions can also enhance the efficiency of electrocatalytic water splitting. Inspired by solar interfacial evaporation, Lu et al. developed a new strategy that simultaneously achieves effective charge transfer and accelerated interfacial gas release, thereby improving interfacial electrocatalytic performance. They integrated perovskite oxide (La_1−x_Sr_x_CoO_3_) with ultrathin 2D Ti_3_C_2_T_x_ nanosheets and constructed an interfacial photothermal evaporation-promoted electrocatalytic system for simultaneous interfacial solar evaporation and water splitting. Compared to pure electrocatalytic water splitting, the introduction of interfacial solar evaporation not only effectively increases the local catalytic temperature but also enhances convection above the catalyst surface, accelerating gas release and escape, and enriching OH^−^ near the catalyst surface, all of which significantly improve catalytic performance [110]. Xu et al. first demonstrated the potential of perovskite oxides as electrocatalysts for hydrogen production in alkaline media, synthesizing a novel perovskite electrocatalyst Pr0.5BSCF that exhibits excellent HER activity and stability under alkaline conditions [20]. Furthermore, for the oxygen evolution reaction (OER) at the anode, the oxygen evolution can be enhanced by increasing the participation of lattice oxygen in perovskite oxides. Pan et al. revealed the contribution of lattice oxygen participation to the OER by designing a model system of silicon-doped strontium cobaltite perovskite electrocatalysts and demonstrated its potential in improving OER activity [111]. Perovskite materials have a promising future in electrocatalytic water splitting and broader energy conversion and storage applications. With continuous advancements in materials science, nanotechnology, and interfacial engineering, the performance of perovskite materials will be further optimized, and their industrial applications will mature and expand.

## 4. Design and Optimization of Photo(electro)catalysis

### 4.1. Catalyst Design and Optimization

Based on the above analysis, the choice of catalyst has a decisive impact on the efficiency, stability, and economy of photo(electro)catalytic water splitting for hydrogen production. Therefore, researching and developing new high-efficiency catalysts is one of the key steps towards the commercial application of this technology. In addition, designing and optimizing photo(electro)catalytic materials to improve the preparation process of water splitting catalysts is also of great significance for achieving an efficient and low-cost hydrogen production process [112,113].

#### 4.1.1. Material Structure

##### Crystal Facet Adjustment

**Figure 10:**
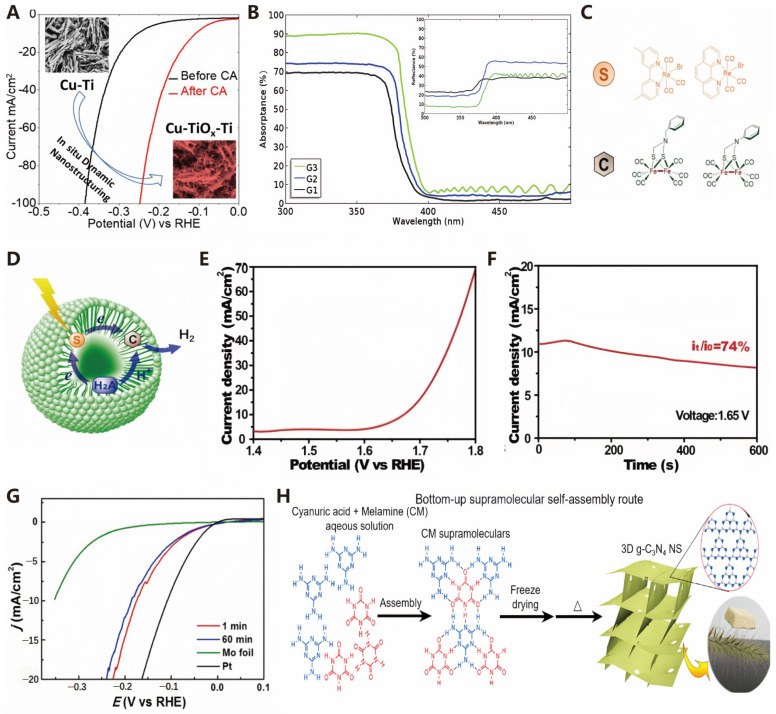
(**A**) CV plots of CuO-Ti electrodes obtained before and after CA for 24h at −0.2 V vs. reversible hydrogen electrode (RHE) (reproduced from [118] with permission from American Chemical Society, copyright 2018). Photocatalytic H_2_ evolution in an aqueous sodium dodecyl sulfate (SDS) micelle solution. (**B**) Optical absorptance spectra of different GaN samples. The inset is optical reflectance spectra (reproduced from [119] with permission from Elsevier, copyright 2019). (**C**) The chemical structures of Re(I) complexes as photosensitizers and [FeFe] H_2_ases as catalysts, S: photosensitizers, C: catalysts. (**D**) The proposed mechanism diagram of the SDS micelle system, H_2_A: ascorbic acid (reproduced from [122] with permission from American Chemical Society, copyright 2010). (**E**) Linear sweep voltammetry of the CoNi(OH)_x_||NiN_x_ nanotube two-electrode water splitting system, and (**F**) chronoamperometry of water electrolysis using the CoNi(OH)_x_||NiN_x_ nanotube system at a voltage of 1.65 V versus RHE (reproduced from [123] with permission from John Wiley & Sons, copyright 2016). (**G**) Cathodic polarization curves of MoS_2_ nanofilms on the Mo foil compared with a pure Mo foil and a standard Pt electrode (reproduced from [129] with permission from American Chemical Society, copyright 2016). (**H**) Schematic illustration of the bottom-up supramolecular self-assembly route for synthesizing 3D g-C_3_N_4_ NS (reproduced from [131] with permission from Elsevier, copyright 2019).

The electrochemical activity is directly related to the nano/macrostructure of the catalyst. It is well known that nanoscale electrocatalysts can increase the surface area and the number of active sites. Engineering materials at the nanoscale, such as regulating the crystal facets of the material, is a common method for optimizing catalytic performance [114]. By controlling the exposed crystal facets with specific atomic arrangements, the physical, chemical, and electronic properties of the electrocatalyst can be optimized [115]. Although high-index crystal facets are difficult to obtain due to their high surface energy, their abundant undercoordinated sites and favorable surface atomic structures can effectively promote catalytic kinetics and control product selectivity [116]. Hellstern et al. [117] explored the performance of two different silicon-based photocathode nanostructure strategies for photochemical hydrogen production: one is to fabricate nanostructures directly on the surface of silicon, and the other is to add nano-structured zinc oxide to increase the electrocatalyst surface area of flat silicon. Their results show that the silicon photocathode supported by nano-structured ZnO has an open circuit voltage about 50 mV higher than that of the nano-structured silicon electrode under one solar intensity light and shows higher electrocatalytic activity. Shinde et al. found that, when copper oxide (CuO) nanoplates are grown on a titanium substrate participate in the HER, not only is CuO reduced to metallic copper (Cu) nanoplates, but both the catalyst and the support undergo a nanocrystallization process. By adjusting the potential, the nanostructure of the catalyst can be precisely controlled, thereby enhancing the catalyst’s activity by 5.4 times. This improvement is not only due to the increase in the active surface area but also benefits from the enhanced water dissociation activity facilitated by the in situ formed TiOx nanoparticles (Figure 10A) [118]. Li et al. developed a single-step, top-down method for preparing nanoporous gallium nitride (GaN) photoelectrodes. The surface nanostructure significantly increases the surface-to-volume ratio of GaN, enabling more incident light to be captured and absorbed by the nanoporous structure. As shown in Figure 10B, the absorptance and reflectance of ultraviolet light for the nanoporous GaN are notably improved, reaching nearly 89% and 9%, respectively [119].

##### Dimension Change

The size of the catalyst has a significant impact on its catalytic performance. Each dimensional structure possesses unique physical and chemical properties that influence its catalytic behavior [114]. Zero-dimensional (0D) photocatalytic systems, with molecular photocatalysts, can be regarded as the simplest prototypes, and they have a long history dating back to the pioneering work by Kirch et al. in the 1970s [120,121]. Wu et al. demonstrated that [FeFe]-hydrogenase (H_2_ase) mimics exhibit exceptional photocatalytic performance for hydrogen production from water splitting. Spherical micelles formed by the self-assembly of amphiphiles in solution are typical high-surface-curvature morphologies and represent a classic example of zero-dimensional (0D) supramolecular materials [122]. In this setup, Re(I) complexes with 4,4′-dimethylbpy or 1,10-phenanthroline ligands, namely, Re(I)(4,4′-dimethylbpy)(CO)_3_Br and Re(I)(1,10-phenanthroline)(CO)_3_Br, were employed as photosensitizers. Meanwhile, [FeFe] H_2_ase mimic catalysts, specifically [Fe_2_(CO)_6_(µ-adt)CH_2_C_6_H_5_] and [Fe_2_(CO)_6_(µ-adt)C_6_H_5_] where µ-adt represents N(CH_2_S)_2_, were used (as shown in Figure 10C,D). Ascorbic acid (H_2_A) served as both a sacrificial electron donor and a source of protons. The efficiency of the HER revealed that the closeness and the hydrophobic interaction were vital in promoting the electron transfer from the excited Re(I) complexes to the [FeFe] H2ase catalysts, thus facilitating H_2_ generation under visible light exposure.

One-dimensional (1D) electrocatalysts, such as nanotubes, nanorods, and nanowires, show great promise due to their high conductivity and structural stability. One-dimensional nanotubes and nanorods have garnered considerable attention in the field of electrocatalysis owing to their high flexibility, excellent conductivity, and inherent anisotropic morphology. Li et al. developed a facile method for synthesizing homologous cobalt-nickel-based nanotube systems directly on conductive copper substrates. As illustrated in Figure 10E,F, when CoNi(OH)_x_ and NiN_x_ nanotubes are combined, the system exhibits excellent complementary water splitting performance and high efficiency. Their work provides a unique approach to designing novel and efficient electrocatalysts for harnessing clean energy [123].

Two-dimensional (2D) electrocatalysts, which appear as sheets, include graphene, nanomembranes, nanolayers, and nano-coatings. Molybdenum sulfide (MoS_2_) is a layered solid rich in rare earth elements, with relatively weak van der Waals forces between the layers and a spacing of about 0.65 nm. Due to its hydrogen binding energy being close to that of platinum-group metals, MoS_2_ exhibits significant HER catalytic activity [124,125,126,127,128]. However, the constraints imposed by the limited number of catalytic active sites, insufficient electrical conductivity, and restricted specific surface area in MoS_2_ nanostructures impede the utilization of MoS_2_-based electrocatalysts for an efficient HER. Hu et al. developed a method for preparing vertically aligned MoS_2_ nanofilms on molybdenum foil to enhance the catalytic activity, conductivity, and specific surface area of MoS_2_-based electrocatalysts in the HER. The MoS_2_ nanofilms, with a thickness of approximately 4 nanometers, were prepared through a two-step process: firstly, oxidizing the surface of the molybdenum foil under low pressure to form a MoO_2_ nanofilm, and, secondly, sulfurizing it at 700 °C for 1 minute to obtain the MoS_2_ nanofilm. As shown in Figure 10G, the MoS_2_ film synthesized at 700 °C for 1 minute has high activity with an onset overpotential of 18 mV. This study also indicates that the method can be extended to the preparation of vertically oriented nanofilms of other two-dimensional atomic layers on metal substrates [129].

Three-dimensional (3D) nanomaterials refer to materials that exceed the nanoscale in all dimensions, with a wide variety of types [114]. The 3D open framework g-C_3_N_4_ can address the stacking and agglomeration issues of 2D g-C_3_N_4_ nanosheets, enhance light absorption, provide multidimensional mass transfer channels, and exhibit excellent photocatalytic performance [130]. Studies have found that 3D porous g-C_3_N_4_ can be prepared through self-assembly (Figure 10H), composed of highly crystalline and ultrathin nanosheets, with MCA supermolecules serving as supports to prevent agglomeration. This material offers a special electron migration pathway, achieving efficient and stable overall water splitting under visible light, with activities approximately 11.8 and 5.1 times those of bulk and nanosheet g-C_3_N_4_, respectively [131].

##### Defect Engineering

**Figure 11:**
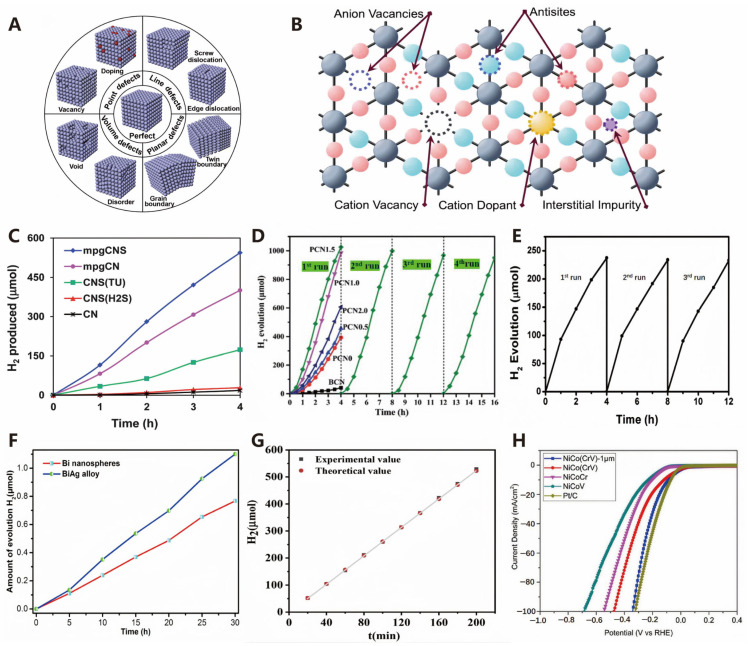
(**A**) Schematic illustrating the defects with different atomic arrangement structures in photocatalytic materials (reproduced from [133] with permission from Elsevier, copyright 2018). (**B**) Schematic illustrating the various point defects in mixed-anion structures with a M^x+^A^y−^B^z−^ crystal representation (M^x+^ = gray, A^y−^ = red, B^z−^ = aqua) (reproduced from [136] with permission from John Wiley & Sons, copyright 2020). (**C**) Hydrogen evolution plot for mpgCNS and control samples (reproduced from [162] with permission from the Royal Society of Chemistry, copyright 2012). (**D**) Photocatalytic hydrogen evolution of some P-g-C_3_N_4_ under visible light irradiation (reproduced from [164] with permission from the Royal Society of Chemistry, copyright 2018). (**E**) Time course of H_2_ evolution of 1at% B-g-C_3_N_4_ nanosheets for 12 h with the evacuation of the system intermittently every 4 h (reproduced from [166] with permission from the Royal Society of Chemistry, copyright 2017). (**F**) photocatalytic H_2_ evolution from aqueous methanol solutions under UV–vis light irradiation over Bi and BiAg alloy nanoparticles (reproduced from [167] with permission from the American Chemistry Society, copyright 2014). (**G**) The theoretical and experimental amount of hydrogen produced by HF-HEA_a2_ as a function of time for 200 min (reproduced from [168] with permission from Elsevier, copyright 2020) [169] (**H**) Cathodic HER performance of MEA thin films (reproduced from [169] with permission from Elsevier, copyright 2024).

Structural irregularities in solid materials can be classified into four types based on their dimensions: zero-dimensional point defects (such as vacancies and dopants), one-dimensional line defects (such as screw dislocations and edge dislocations), two-dimensional planar defects (such as grain boundaries and twin boundaries), and three-dimensional volume defects (such as lattice disorder and voids) [132]. As shown in Figure 11A, these defects can be further subdivided in photocatalytic materials [133]. When preparing nanomaterials, defects are inevitable and can sometimes serve as active sites [134]. Currently, defect engineering methods are emerging endlessly [135] and represent an effective strategy for improving the photocatalytic performance of mixed anion semiconductors. Defects in mixed anion semiconductors include point defects (such as vacancies, interstitials, and antisite defects as illustrated in Figure 11B) [136] as well as higher-dimensional defects. Researchers have recently paid particular attention to vacancies [137], structure–activity relationships [138], and the influence of synthesis conditions on morphology [139]. Oxygen vacancies are common in metal oxide semiconductor photocatalysts, such as TiO_2_ [140,141,142], Bi_2_WO_6_ [143,144], BiOCl [145,146,147], and SrTiO_3_ [148]. Other anion vacancies have also been developed in ionic compound semiconductor photocatalysts, such as sulfur vacancies in ZnS [149]. In addition, there are cation vacancies, such as bismuth [150] and titanium vacancies [151,152]. The covalent compound semiconductor photocatalyst graphitic C_3_N_4_ (g-C_3_N_4_) can generate nitrogen [153,154] and carbon vacancies [155,156].

The role of defects in photocatalysis is diverse, enhancing light absorption, regulating charge behavior, and adjusting surface reactions, which are crucial for improving efficiency. The impact of defects is influenced by their type, position, and quantity, and can be analyzed through technical means [133]. The rational design of defects can optimize various steps of photocatalysis, thereby improving efficiency. Despite progress in defect engineering, challenges remain, particularly in how to balance the positive and negative effects of defects to maximize their advantages.

#### 4.1.2. Composition Optimization

##### Doping

Due to the intrinsic differences in atomic radius, electronic configuration, and electronegativity between the dopant atom and the substituted atom, heteroatom doping can modify the original lattice, induce local electron redistribution, and adjust the d-band center of the catalyst [157]. Furthermore, these changes can significantly enhance the electrocatalytic activity of the catalyst [158].

Taking graphitic carbon nitride (g-C_3_N_4_) as an example for exploration, elemental doping is a simple yet effective modification method widely used in the optimization of g-C_3_N_4_ materials. Due to its inherent band gap, pure g-C_3_N_4_ primarily absorbs visible light. By doping with elements, the band gap and positions of its conduction band (CB) and valence band (VB) can be significantly adjusted. Furthermore, the introduction of a small amount of dopant atoms can also markedly alter the charge distribution and physical properties of g-C_3_N_4_. This method is not only easy to operate but also significantly improves the material’s performance [159,160]. Doping technology has been further expanded by introducing different types of heteroatoms into g-C_3_N_4_. Researchers have conducted in-depth studies on cationic and anionic doping, single-element doping, and composite doping with multiple elements and have thoroughly explored their doping mechanisms [161].

Doping with nonmetallic elements such as sulfur (S), phosphorus (P), oxygen (O), boron (B), nitrogen (N), and halogens (F, Cl, Br, I), which possess high ionization energies and electronegativities, can effectively enhance photocatalytic performance (Table 2). (1) Hong et al. synthesized sulfur-doped mesoporous graphitic carbon nitride (S-doped mpg-C_3_N_4_) through a facile calcination process using thiourea and silicon dioxide (SiO_2_) nanoparticles. This material exhibits a high quantum efficiency of 5.8% at a wavelength of 440 nm, making it an efficient photocatalytic material for hydrogen production. As shown in Figure 11C, in the presence of triethanolamine (TEA), the photocatalytic hydrogen production performance of S-doped mpg-C_3_N_4_ is enhanced by 30 times compared to pure g-C_3_N_4_, while its specific surface area increases by only 10 times [162]. (2) Additionally, Yang et al. successfully designed and synthesized flower-like porous P-doped g-C_3_N_4_ using phosphoric acid as the phosphorus source and a cyanuric acid–melamine complex as the precursor, through an economical and template-free thermal condensation method. This material exhibits better utilization of visible light, achieving a hydrogen production rate of 256.4 μmol h^−1^, which is 24 times that of pure g-C_3_N_4_ (Figure 11D) [163]. (3) Oxygen-doped g-C_3_N_4_ retains its structure while altering its texture and morphology, narrowing the band gap, enhancing light absorption, and accelerating heterogeneous photocatalytic reactions by increasing the surface area [164]. Recently, She et al. successfully prepared two-dimensional porous oxygen-doped g-C_3_N_4_ ultrathin nanosheets using a simple calcination and room-temperature chemical oxidation method, with bulk g-C_3_N_4_ as the raw material and H_2_SO_4_ and HNO_3_ as oxidants [165]. Due to the greater electronegativity of oxygen atoms compared to nitrogen atoms, the interlayer interaction is enhanced after doping, leading to a reduction in interlayer spacing and thus increasing catalytic activity. (4) Recently, Thaweesak et al. reported a novel yellow porous boron-doped g-C_3_N_4_ nanosheet [166]. This nanosheet not only increases the number of surface-active sites but also shortens the diffusion length of charges from the bulk to the surface, effectively reducing the charge recombination rate. Under visible light irradiation, this photocatalyst exhibits significantly enhanced hydrogen generation activity, reaching 1880 μmol g^−1^h^−1^, and demonstrates good stability and reusability. Its hydrogen generation activity is 12 times that of pure g-C_3_N_4_ samples (Figure 11E). (5) Halogen atoms (F, Cl, Br, I) doped into g-C_3_N_4_ exhibit excellent photocatalytic performance due to their enhanced light absorption capability. As the atomic number of the halogen atoms increases, their electronegativity decreases, promoting more electrons to transfer from the halogen atoms to g-C_3_N_4_ and facilitating electron transfer [161]. This doping strategy effectively enhances the photocatalytic performance of g-C_3_N_4_, broadening its application prospects in the fields of energy and the environment.

##### Alloying

Electrocatalytic water splitting is an important hydrogen production technology, with the core focus on developing efficient electrocatalysts to reduce the reaction overpotential and enhance energy conversion efficiency. Alloying represents an effective strategy for improving catalyst performance, as the introduction of a second metal alters the electronic structure and surface properties of the catalyst, thereby enhancing its activity and stability. Jiao et al. found that the formation of the BiAg alloy through the incorporation of Ag significantly enhanced the photoelectrochemical activity of Bi, and the material’s photoelectrochemical performance reached its optimum when the molar ratio of Bi to Ag was 7:3 (Figure 11F) [167]. In addition to the advantages of these elemental semiconductors, both Bi and Ag are heavy elements with greater mass compared to non-metallic materials, making them easily recoverable through precipitation methods in future practical applications.

Given that most electrocatalysts rely on conductive substrates, it is imperative to develop bulk metallic-based catalysts with self-supporting structures, excellent conductivity, and adjustable sizes [168]. Medium-entropy and high-entropy alloys (MEAs and HEAs), unlike traditional alloy approaches, utilize a significant proportion of elements. This alloying technique has garnered considerable interest worldwide for various applications, particularly in electrocatalysis. (1) High-entropy alloys (HEAs) are alloys composed of five or more metals in equal or nearly equal molar ratios, exhibiting unique properties [170]. Many HEAs incorporate Co, Fe, and Ni elements together with corrosion-resistant metals like Cr and Al, indicating their potential as electrocatalysts for HERs in acidic environments [171,172]. Furthermore, their high-entropy configuration, atomic chemical disorder, lattice distortion, and other unique properties endow them with the potential to serve as excellent electrocatalysts. Ma et al. reported a self-supported CoCrFeNiAl high-entropy alloy electrocatalyst for the HER in acidic environments [168]. As shown in Figure 11G, the theoretical and experimental amounts of hydrogen produced by HF-HEA_a2_ over 200 minutes are plotted as a function of time, demonstrating good HER activity. (2) Medium-entropy alloys (MEAs) are a class of alloys with moderate atomic entropy, featuring a more complex atomic composition than traditional alloys but simpler than high-entropy alloys, with entropy values falling between the two. The CoNiCr and CoNiV systems, composed of three elements, feature low hydrogen diffusion rates, strong corrosion resistance, high conductivity, and simple crystal structures, making them ideal materials for HER and OER research [173,174]. As shown in Figure 11H, the electrochemical characterization of MEA films was conducted in a 1 M KOH alkaline medium and compared with a Pt/C catalyst. The cathodic HER polarization curves indicate that NiCo(CrV) outperforms other MEA catalysts [169].

#### 4.1.3. Surface Modification

##### Co-Catalyst Decoration

**Figure 12:**
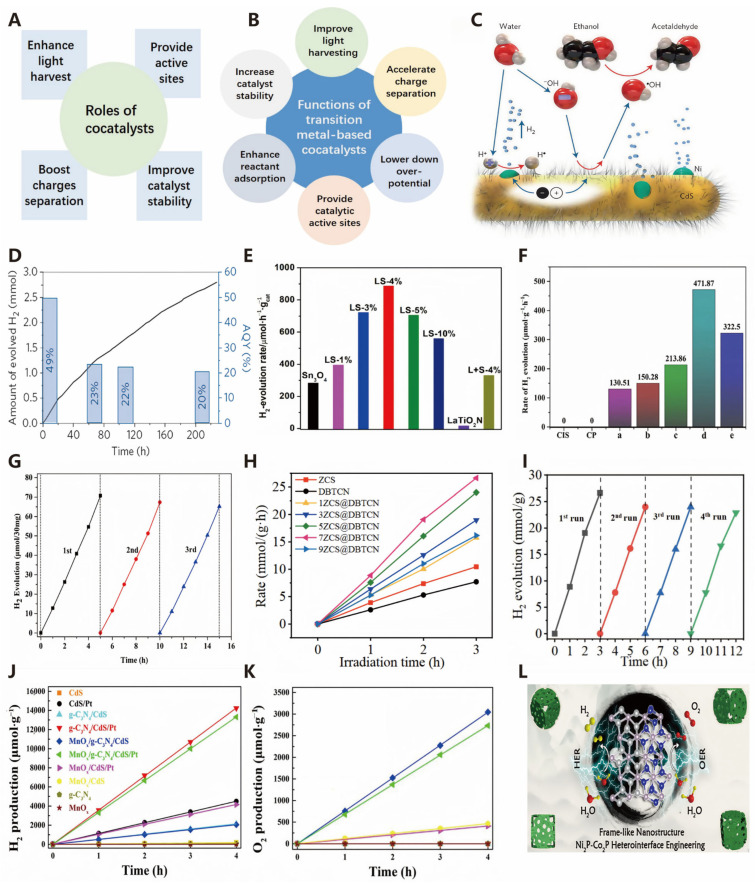
(**A**) Functions of cocatalysts in photocatalytic H_2_ production (adapted from [199] with permission from Elsevier, copyright 2020). (**B**) Functions of transition metal-based cocatalysts in photocatalytic water splitting (adapted from [181] with permission from John Wiley & Sons, copyright 2022). (**C**) Schematic illustration of the photocatalytic H_2_ generation mechanism over Ni-CdS [183]. (**D**) Long-term evolution of H_2_ over versus illumination time with apparent quantum efficiency (AQEs) at 447 nm (the bars) in the Ni-CdS photocatalytic system (reproduced from [182] with permission from Springer Nature, copyright 2014). (**E**) Photocatalytic H_2_-evolution rates of as-prepared LaTiO_2_N/Sn_3_O_4_ samples under visible-light irradiation (λ > 400 nm) (reproduced from [190] with permission from Elsevier, copyright 2024). (**F**) The average H_2_-evolution rate for all photocatalysts (a: CIS-Pt; b: 5CPCIS; c: 10CPCIS; d: 15CPCIS; e: 20CPCIS). (**G**) Stability test of 15CPCIS heterojunction composites (reproduced from [191] with permission from Elsevier, copyright 2023). (**H**) PHE rates of DBTCN, ZCS, and ZCS@DBTCN samples. (**I**) Stability test of PHE over 7ZCS@DBTCN (reproduced from [193] with permission from Elsevier, copyright 2023). The photocatalytic performances of different samples, (**J**) the HER performance (half-reaction, Na_2_S/Na_2_SO_3_ as hole sacrificial agent), (**K**) the OER performance (half-reaction, AgNO_3_ as electron sacrificial agent) (reproduced from [194] with permission from Elsevier, copyright 2021). (**L**) Frame-like nanostructure Ni_2_P-Co_2_P Heterointerface (reproduced from [195] with permission from the American Chemical Society, copyright 2021).

In the photocatalytic hydrogen production system, co-catalysts play four key roles in enhancing the activity of semiconductor photocatalysts (Figure 12A), namely, enhancing light harvesting capability [175,176], promoting the separation and transport of charge carriers [177,178], providing proton reduction sites [179,180], and improving the stability of photocatalysts. As shown in Figure 12B, the loading of transition metal-based co-catalysts exerts various effects in enhancing the photocatalytic performance of semiconductor photocatalysts in photocatalytic water splitting [181]. Feldmann et al. deposited nickel nanoparticles with sizes ranging from 2 to 8 nanometers on cadmium sulfide nanorods via a photo-deposition method (Figure 12C), achieving an optimal photocatalytic hydrogen production rate of 63 mmol g^−1^h^−1^ at a wavelength of 477 nanometers, with an apparent quantum efficiency (AQE) of 53% (Figure 12D) [182].

##### Heterojunction Construction

Heterojunction catalysts play a significant role among emerging catalysts. Their electrons rearrange at the interface to modify active sites, and the synergistic effects of different sites promote reaction kinetics [183,184]. The advantages include the following: interfacial lattice strain exposes more active sites, enhancing efficiency; diverse morphological designs [185] can increase specific surface area and active sites; charge transfer or complementary redox properties between components enhance reaction activity and efficiency [186,187,188,189].

Heterojunction catalysts play a crucial role in the process of photocatalytic water splitting. They are composed of two different semiconductor materials combined through surface or point contact. This combination helps to reduce the recombination of electron–hole pairs while enhancing their separation efficiency [3]. (1) Zong et al. successfully constructed a LaTiO_2_N/Sn_3_O_4_ heterojunction with a type-II structure and hierarchical architecture via a hydrothermal method. Compared to pure Sn_3_O_4_ and LaTiO_2_N, this heterojunction exhibits approximately 3-fold and 52-fold enhancements in photocatalytic hydrogen production activity, respectively, achieving a hydrogen production rate of 887 μmol g_cat_^−1^h^−1^(Figure 12E). The introduction of LaTiO_2_N enables Sn_3_O_4_ to photocatalyze water splitting using 600 nm visible light [190]. (2) Ma et al. prepared a heterojunction composed of CdIn_2_S_4_ (CIS) nanoparticles and Co_2_P (CP) through a hydrothermal method. The light absorption range of this CPCIS heterojunction extends across the entire visible light region, with significantly enhanced absorption intensity, particularly in the wavelength range of 500 to 800 nm. The hydrogen production rate of 15CPCIS reaches 471.87 μmol g^−1^h^−1^, which is approximately 3.6 times that of CIS-Pt (Figure 12F). After three cycles, the photocatalytic activity of the heterojunction composite remains stable (Figure 12G) [191]. (3) Studies have shown that the covalent-bonded Z-scheme heterojunction structure can enhance visible light absorption and improve the separation and rapid transfer of photogenerated carriers. Chen et al. synthesized a covalent-bonded doped carbon nitride/graphitized carbon nitride hybrid (O-CN/CN) through solvothermal treatment. As shown in Table 3, under visible light irradiation, the hydrogen production rate of O-CN/CN-3 is 12.4 times that of the pristine CN, reaching 6.97 mmol g^−1^h^−1^, and the activity remains essentially unchanged after five cycles [192]. (4) The synergistic effect of the intramolecular electric field and the interfacial S-scheme heterojunction can greatly accelerate the intramolecular and interfacial transfer of photoexcited carriers, maintaining the maximum redox capabilities of spatially separated electrons and holes. Li et al. synthesized the ZCS@DBTCN S-scheme heterojunction photocatalyst through a self-assembly method. 7ZCS@DBTCN exhibits optimal hydrogen production performance, reaching 8.87 mmol g^−1^h^−1^, which is 2.55 times and 3.46 times that of pure ZCS and DBTCN, respectively (Figure 12H). Furthermore, the hydrogen production rate of 7ZCS@DBTCN remains essentially unchanged after four cycles, demonstrating good stability (Figure 12I) [193]. (5) Core–shell structures can enhance structural stability, thereby improving photocatalytic stability and favoring the enhancement of photocatalytic performance. For example, Pan et al. prepared a MnOx/g-C_3_N_4_/CdS/Pt hollow core–shell heterojunction through a sequential chemical annealing-photoreduction method. This hollow core–shell heterojunction exhibits significantly enhanced performance in the HER, approximately 80 times that of pristine CdS, with an overall water splitting performance of 1303.39 (H_2_) or 641.60 (O_2_) μmol g^−1^h^−1^ (Figure 12J,K). Additionally, the spherical structure improves physical stability, while the thin heterojunction inhibits photocorrosion, thereby enhancing stability [194].

In addition, heterojunction catalysts often exhibit better electrocatalytic water splitting activity than single-component catalysts, making them extensively studied in the field of electrocatalytic water splitting. (1) By constructing heterojunctions, electron redistribution can occur at the interfaces, achieving synergistic effects and thus enhancing the catalyst’s activity. For instance, Lvlv Ji et al. [195] synthesized a novel frame-like nanostructured catalyst, (Ni, Co)_2_P nano-frames (NFs) (Figure 12L), through precipitation, chemical etching, and phosphorylation steps. This catalyst, composed of Ni_2_P−Co_2_P nanoparticles embedded in a doped carbon matrix with a heterojunction structure, serves as a bifunctional catalyst for both the HER and OER, outperforming single-component Ni_2_P, Co_2_P samples, and (Ni, Co)_2_P solid nano-cubes. Through a combination of systematic experiments and theoretical calculations, the mechanism of Ni_2_P-Co_2_P heterojunction regulation optimizing the catalyst’s intrinsic hydrogen evolution activity was revealed, and the structural advantages of the nano-frame in increasing the number of catalytic active sites and enhancing mass transfer and diffusion capabilities were elucidated. (2) Heterojunction catalysts can improve the stability of catalysts during the water splitting process. For example, NiCo bimetallic single-atom catalysts (Ni Co bim SAC) exhibit excellent stability in both acidic and alkaline media, with no significant performance degradation observed [196]. (3) In heterojunctions, the different energy band alignments of the various phases lead to charge transfer at the interfaces, which is beneficial for surface electron modulation of the heterojunction, thereby promoting charge separation. For instance, by constructing the CoFeOOH catalyst with high-valent metal sites and amorphous/crystalline interfacial heterojunctions, the transformation of Co (II) to Co (III) high-valent metal sites during the electrochemical reconstruction process can be promoted, facilitating the regulation of the catalyst’s active components [197]. (4) Heterojunction catalysts can reduce energy consumption during the hydrogen production process via water splitting. For example, in the multi-dimensional core–shell structured Ni/NiCoP heterojunction catalyst, there is a strong electron transfer from the Ni core to the bimetallic NiCoP, resulting in a special electronic structure effect. This strong electronic structure effect effectively promotes the water splitting reaction [198].

**Table 3:** Comparisons of graphitic CN-based photocatalysts with assistance of 3 wt% Pt for H_2_ generation using 300 W xenon lamp.

Photocatalysts	Light Source	H_2_ generation Rate (μmol g^−1^h^−1^)	Ref.
O-doped CN/TiO_2_	λ > 400 nm	587	[200]
CoTiO_3_/CN	λ > 420 nm	858	[201]
FeO_x_/CN	780 > λ > 420 nm	1080	[202]
S-CN/CN	λ > 420 nm	4765	[203]
CN/CoO	λ > 400 nm	651	[204]
p-n homojunction CN	λ > 400 nm	4020	[205]
O-CN/CN	λ > 420 nm	6970	[192]

### 4.2. Improvement of Photo(electro)catalysis System

#### 4.2.1. pH of Reaction System

**Figure 13:**
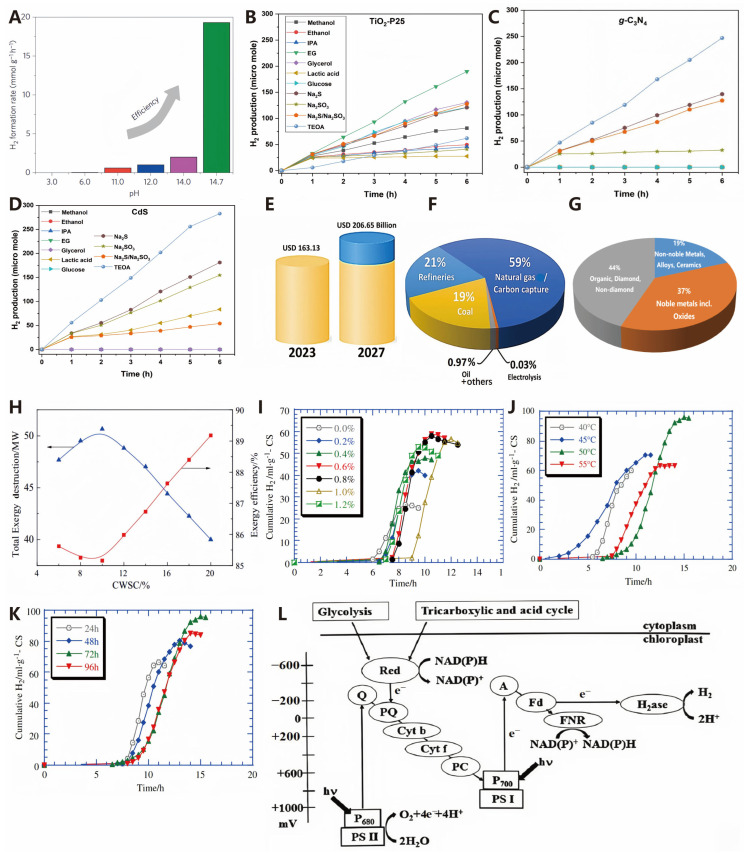
(**A**) Effect of pH of the CdS nanocrystal dispersion. Comparison of H_2_ formation rates over 6 h showing strong enhancement at high pH (reproduced from [182] with permission from Springer Nature, copyright 2014). Photocatalytic H_2_ production efficiency of (**B**) TiO_2_-p25, (**C**) g-C_3_N_4_, and (**D**) CdS using various sacrificial agents [206]. (**E**) Global hydrogen market projection in terms of value. (**F**) Comparison of hydrogen production sources. (**G**) Materials as electrocatalysts for water splitting. (reproduced from [196] with permission from Springer Nature, copyright 2024). (**H**) Total exergy destructions and exergy efficiencies in different CWSCs (reproduced from [207] with permission from Elsevier, copyright 2018). Effect of enzymatic (**I**) pH, (**J**) temperature, or (**K**) time on bio-H_2_ production (reproduced from [208] with permission from Elsevier, copyright 2010). (**L**) Diagram of hydrogen production mechanism of green algae (reproduced from [209] with permission from Oxford University Press, copyright 2001).

The activity of a catalyst is frequently tightly linked to its surface charge condition, and variations in pH have a direct impact on the distribution of charge on the catalyst’s surface. Therefore, adjusting the pH can optimize the active sites of the catalyst, enhancing its adsorption capacity for reactants and desorption capacity for products. For example, in some photocatalytic systems, an appropriate pH can make the active sites on the catalyst’s surface more easily bind with reactants, thereby promoting the progression of the photocatalytic reaction. Different pH values may alter the reaction pathway, making the reaction more favorable for generating hydrogen or other desired products. By adjusting the pH, the reaction can be guided along a more favorable path (Figure 13A).

#### 4.2.2. Addition of Sacrificial Agents

In the photocatalytic hydrogen generation process, the segregation and recombination of light-induced electrons and holes play crucial roles in determining the hydrogen production efficiency. The rejoining of holes leads to the consumption of light-induced electrons, consequently lowering the efficiency of hydrogen generation. The addition of a sacrificial agent can effectively consume holes, reducing their recombination with electrons and thus improving the utilization efficiency of electrons and the efficiency of hydrogen production. Commonly used sacrificial agents include methanol, ascorbic acid, and triethanolamine. These sacrificial agents can react with holes to generate harmless or further utilizable products, thereby avoiding the accumulation of holes. The addition of sacrificial agents can also alter the kinetic behavior of the reaction. Some sacrificial agents can interact specifically with reactants or catalysts; reducing the activation energy of the reaction or altering the constant of the reaction rate can expedite the progression of the hydrogen production reaction. For instance, the efficiencies of hydrogen generation for three promising photocatalytic materials, TiO_2_-P25 (Figure 13B), g-C_3_N_4_ (Figure 13C), and CdS (Figure 13D), were evaluated in detail using different sacrificial agents [206].

In practical applications, it is often necessary to combine multiple optimization strategies, such as adjusting the pH value and adding sacrificial agents, to achieve the best hydrogen production results. For example, in a specific photocatalytic hydrogen production system, the optimal pH range is first determined through experiments to maximize the catalyst’s activity. Then, under these pH conditions, an appropriate amount of sacrificial agent is added to consume holes and optimize the reaction kinetics. At the same time, other factors such as light source intensity, catalyst dosage, and reaction temperature can also be considered, and comprehensive optimization can be implemented to achieve the maximum hydrogen production efficiency.

#### 4.2.3. Synergistic Effects Between Catalyst and Electrode

Catalysts and electrodes each play crucial roles in electrochemical reactions, and their synergistic effect can significantly enhance the efficiency and performance of these reactions. This synergy can be achieved through various means, including but not limited to enhancing the quantity of active sites, boosting the inherent activity of each active site [210], modifying the electronic configuration of active sites [211], and optimizing the interaction between the electrode surface and the catalyst [212]. The development and application of these strategies provide important theoretical foundations and practical guidance for the creation and advancement of innovative and high-performance electrocatalysts. By deeply understanding the mechanism of the synergistic effect between catalysts and electrodes, we can further optimize electrocatalytic reactions, improve energy conversion efficiency, and provide a scientific basis for addressing energy and environmental issues.

## 5. Economics of Hydrogen Production Technologies

According to the latest research, the global market size for green hydrogen is expected to grow significantly in the coming years, from the current USD 16.313 billion to USD 206.65 billion (Figure 13E) [213,214,215]. Green hydrogen is primarily produced from various resources, including fossil fuels, biomass, and water. Currently, natural gas serves as the main feedstock for hydrogen generation (Figure 13F). However, the process of water splitting, wherein water is broken down into hydrogen and oxygen, presents a more straightforward, efficient, and eco-friendly approach to hydrogen production, with the capability to supplant conventional fossil fuel-dependent methods [216]. Although scientists have made some progress in searching for more efficient hydrogen production catalysts, precious metal-based nanoparticle catalysts currently remain the most efficient choice for hydrogen production (Figure 13G) [217,218]. To assess the economics of hydrogen production technologies and determine future development directions, we will evaluate several major hydrogen production technologies. Through these analyses, we can gain a clearer understanding of the advantages and disadvantages of different hydrogen production technologies, thereby providing guidance for future research and investment decisions.

### 5.1. Water Splitting for Hydrogen Production

Water is among the most plentiful renewable resources available on Earth. Through the process of water decomposition, hydrogen can be obtained, and, upon combustion, hydrogen converts back into water. As a result, water can be reused in this cycle without generating any harmful by-products, making it an ideal choice for hydrogen production.

#### 5.1.1. Photocatalytic Hydrogen Production

The economic viability of hydrogen generation through photocatalysis from water decomposition is influenced by multiple factors: (1) Performance of photocatalysts: The efficiency of photocatalysts directly impacts the expense of hydrogen generation. High-efficiency photocatalysts can reduce the production cost per unit of hydrogen. For instance, the silicon nanowire materials developed by the research team led by James F. Cahoon are capable of decomposing water to produce hydrogen under visible and infrared light. The innovative design of these materials enhances the utilization and conversion efficiency of light energy, contributing to lower hydrogen production costs [219]. (2) Stability and lifespan of photocatalysts: The stability and service life of photocatalysts are also crucial factors affecting economics. A stable photocatalyst can maintain high catalytic activity over a long period, thereby reducing replacement and maintenance costs. The catalytic performance and stability of some photocatalytic materials can be found in Table 4. (3) Efficiency of light source utilization: During the photocatalytic generation of hydrogen, the efficiency of light source utilization also affects economics. Improving the utilization efficiency of sunlight or developing photocatalysts that can work efficiently under various lighting conditions will help reduce hydrogen production costs. (4) Material costs: The material costs of photocatalysts are also an important consideration. Using inexpensive and abundant materials can significantly lower the production costs of photocatalysts.

#### 5.1.2. Electrocatalytic Hydrogen Production

The commercial application of water electrolysis first emerged in the 1890s. Nevertheless, despite over a century of development, electrochemical water splitting still contributes to just 4% of the world’s hydrogen production [233]. In real-world applications, electrolysis systems exhibit energy conversion efficiencies ranging from roughly 56% to 73% [234], which are relatively modest and severely restrict their widespread adoption. From the vantage point of sustainable energy progression, electrocatalytic water splitting possesses immense potential for the extensive use of green hydrogen energy and is of paramount importance in practical implementation. The creation of electrolysis water catalysts that demonstrate outstanding catalytic performance, high durability, and cost-effectiveness is vital for the realization of green energy and sustainable progress. As shown in Table 5, several key parameters for evaluating catalyst performance are listed. The overpotential at 10 mA/cm^2^ reflects the catalyst’s activity. The exchange current density represents the charge exchange rate, with a higher value indicating better activity. The Tafel slope characterizes the reaction kinetics. The electrochemical active surface area (ECSA) reflects the actual active surface area. The stability parameter is used to assesses the durability performance. These parameters collectively provide a basis for evaluating catalyst performance.

### 5.2. Hydrogen Production from Fossil Fuels

In hydrogen production technologies, fossil fuel-based hydrogen production is a method that primarily uses coal and natural gas as raw materials. Specifically, coal-based hydrogen production entails the process of reacting coal with water under elevated temperatures and pressures to produce hydrogen and carbon monoxide, which is subsequently followed by a reaction where carbon monoxide reacts with water to yield additional hydrogen. Natural gas-based hydrogen production consists of two steps. Firstly, there is the steam reforming process, in which natural gas is combined with steam at temperatures between 700 °C and 850 °C to generate hydrogen and carbon monoxide. Secondly, the water–gas shift reaction takes place, wherein carbon monoxide interacts with water to produce additional hydrogen. These methods can yield high-purity hydrogen with conversion efficiencies reaching 70% to 80%. However, due to the need for high-temperature and high-pressure conditions, these processes consume significant amounts of energy, and elements such as sulfur and phosphorus in coal can produce toxic gases during gasification. To obtain high-purity hydrogen, the product must be purified, further increasing the cost of hydrogen production. Zhe et al. developed a supercritical water gasification technology, wherein the lowest temperature within the gasifier is achieved at varying coal–water slurry concentrations (CWSCs). As shown in Figure 13H, as the CWSC increases, the total exergy loss first rises and then falls. The highest exergy loss is seen when the CWSC is around 10%. In contrast, exergy efficiency displays a trend that is the reverse of the total exergy loss. The process reaches its peak exergy efficiency of 89.18% at a CWSC of 20%. This technology enables hydrogen production at lower temperatures and allows elements such as sulfur and phosphorus to dissolve in supercritical water in the form of inorganic salts, significantly reducing the cost of hydrogen production [207]. Nevertheless, this hydrogen production method still requires a substantial amount of fossil fuels, which is inconsistent with the concept of sustainable energy development.

### 5.3. Hydrogen Production from Bioenergy

Hydrogen production from bioenergy encompasses microbial hydrogen production technologies, as well as biomass pyrolysis and gasification processes [209]. Microbial hydrogen production relies on the metabolic activities of microorganisms to generate hydrogen. Currently, this method primarily depends on anaerobic bacteria, photosynthetic bacteria, and green algae for hydrogen generation. Anaerobic bacterial hydrogen production occurs in an oxygen-free and light-free environment, where anaerobic bacteria decompose organic matter to release hydrogen. The anaerobic fermentation process is significantly influenced by factors such as pH, temperature, and fermentation time. For instance, Yu et al. explored the impact of different enzymatic hydrolysis pH values (Figure 13I), enzymatic hydrolysis temperature (Figure 13J), and enzymatic hydrolysis time (Figure 13K) on hydrogen production from the anaerobic fermentation of corn stover waste [208]. On the other hand, green algae produce hydrogen by directly splitting water molecules into hydrogen and oxygen (Figure 13L) [209]. Taken together, biological hydrogen production typically requires anaerobic or illuminated conditions and is susceptible to environmental changes, with relatively low efficiency. Therefore, it is not well suited for large-scale hydrogen production.

Hydrogen production through biomass pyrolysis is a technology that involves heating and decomposing biomass feedstocks under oxygen-deficient or limited oxygen conditions to generate hydrogen and other by-products (such as CO, bio-oil, and biochar). Subsequently, the hydrogen is purified through filtration to obtain high-purity hydrogen [246]. Unlike pyrolysis, bio-gasification for hydrogen production requires the introduction of certain gasifying agents (such as steam, oxygen, etc.) during the process [247]. Compared to biological hydrogen production methods, biomass pyrolysis and gasification offer higher efficiency and yield hydrogen with higher purity (40–60%). However, prior to hydrogen production via pyrolysis and gasification, lignin must be removed from materials like wood chips and rice husks, which increases the cost of hydrogen production [247,248]. Moreover, these two hydrogen production technologies generate significant amounts of tar, leading to pipeline blockages [246], which greatly impact subsequent processing and limit their large-scale industrial application.

Apart from the aforementioned three hydrogen production methods, hydrogen can also be produced from waste plastics. The development of this technology not only addresses waste disposal issues but also yields valuable energy, aligning with the concept of green development. In the process of the catalytic pyrolysis of waste plastics, aromatic hydrocarbons and carbon nanotubes (CNTs) are produced, along with a considerable amount of hydrogen in the gaseous products [249]. Liu et al. [250] discovered that a notable quantity of hydrogen is generated during the synthesis of carbon nanotubes using Ni-based catalysts, and this production is closely linked to temperature. The hydrogen content peaked at 77 vol%, with a carbon nanotube yield of 376 mg per gram of plastic. Yao et al. enhanced the hydrogen production by introducing steam into the catalytic reforming phase. As the steam-to-plastic ratio rose from 0 to 2.6, the hydrogen yield climbed from 31.8 to 92.7 mmol H_2_ per gram of plastic, albeit at the expense of a 3.5 wt% reduction in CNT yield [251]. Nevertheless, the technology for hydrogen production through the catalytic pyrolysis of waste plastics remains in the research stage, and numerous technical hurdles, including catalyst deactivation caused by carbon deposition, must be surpassed [252].

Table 6 illustrates the economic comparison of various hydrogen production technologies [253,254,255]. In summary, hydrogen production holds promise as the most economically efficient means of converting syngas into transportation fuels. Electrocatalytic water splitting currently stands as the only method capable of large-scale hydrogen production without generating traditional fossil fuel byproducts. Biomass-based hydrogen production technologies, including biomass gasification, pyrolysis, and fermentation, are still in their developmental stages. These technologies offer the potential to extract hydrogen from waste materials such as cellulosic biomass and wastewater. Solar energy can be harnessed to produce hydrogen through thermochemical, photochemical, or electrochemical pathways. Photocatalytic water splitting may represent the most efficient method for extracting hydrogen from water, as it bypasses the inefficient step of converting solar energy into electricity for electrolysis.

## 6. Conclusions and Prospects

Hydrogen energy, owing to its high energy density and clean combustion characteristics, is regarded as a crucial option for future energy sources. This paper reviews the scientific principles and recent progress of photo(electro)catalytic water splitting for hydrogen production. It summarizes the fundamental principles of photo(electro)catalytic water splitting, including the mechanisms of photocatalytic reactions and electrocatalytic hydrogen and oxygen evolution reactions. Furthermore, it provides a detailed analysis of the latest research progress in various photo(electro)catalytic materials, such as TiO_2_, metal sulfides, metal nitrides and nitrides, and novel photocatalysts. Additionally, this paper explores strategies for optimizing photo(electro)catalytic materials, including material structural design, composition optimization, and surface modification. In terms of optimizing photo(electro)catalytic materials, it is pointed out that strategies such as nanoscale facet adjustment, dimensionality adjustment, defect engineering, doping, alloying, and surface modification can significantly enhance catalyst performance. These strategies not only improve the separation efficiency of photogenerated electron–hole pairs but also enhance the stability and activity of the catalysts. This paper also discusses the importance of improving reaction conditions (such as adjusting pH and adding sacrificial agents) for enhancing photo(electro)catalytic performance and compares the economics of different hydrogen production technologies.

Despite significant progress in photo(electro)catalytic water splitting for hydrogen production, numerous challenges still lie ahead. Further exploration is needed in the following areas: (1) Development of efficient and stable photo(electro)catalysts: The search for novel photo(electro)catalyst materials, particularly those with visible light response and efficient electron–hole separation capabilities, should continue [256,257,258]. By adjusting the energy band structure, surface properties, and interfacial effects of the materials, the activity and stability of the catalysts can be further enhanced [259]. (2) Optimization of the photo(electro)catalytic system: Research should focus on the integration and optimization of the photo(electro)catalytic system, including the selection of light sources, the design of reactors, and the synergistic interaction between catalysts and electrodes. By optimizing the system structure, energy conversion efficiency and hydrogen production rate can be improved [256,260]. (3) Reduction of hydrogen production costs: Efforts should be made to explore low-cost, easily prepared photo(electro)catalyst materials, as well as to increase the reuse cycles and stability of the catalysts, thereby reducing hydrogen production costs. Additionally, combining photo(electro)catalytic water splitting with other renewable energy technologies, such as solar and wind energy, should be explored to achieve more efficient energy utilization [261,262,263]. (4) Promotion of industrial applications: Research on the industrialization of photo(electro)catalytic water splitting for hydrogen production should be strengthened, including the large-scale preparation of catalysts, the scale-up design of reactors, and the integration and testing of systems. By integrating with actual industrial applications, the commercialization process of this technology can be promoted [264,265]. (5) Addressing environmental issues: While advancing the development of photo(electro)catalytic water splitting for hydrogen production, attention should also be paid to its environmental impact. Research should focus on the recovery and reuse of catalysts to reduce environmental pollution. Simultaneously, exploring the resource utilization of by-products generated during the hydrogen production process can help achieve green and sustainable energy production [266,267]. In summary, photo(electro)catalytic water splitting holds great potential, and it is expected to solve the current energy and environmental challenges, driving the sustainable development in the future.

## Figures and Tables

**Table 1:** Hydrogen evolution effect of TiO_2_ composite photocatalysts.

Catalyst	Sacrificial Agent	Exposure Condition	HER (μmol∙g^−1^∙h^−1^)
Cu_3_Mo_2_O_9_/TiO_2_	TEOA	300 W xenon lamp	3401.90
NiO/TiO_2_	methyl alcohol	300 W xenon lamp	228.00
TiO_2_@SiO_2_	methyl alcohol	Ultraviolet ray	410.61
Cu_2_O/TiO_2_	methyl alcohol	100 MW/cm^2^ xenon lamp	11,000.00

**Table 2:** Summary of g-C_3_N_4_ based photocatalysts doped with different heteroatoms.

Photocatalyst	Dopants and Fabrication Methods	Activity Enhancement (Higher than Pure g-C_3_N_4_)	Ref.
S, SiO_2_-CN	thiourea and SiO_2_ nanoparticles, simple calcination	30 times	[162]
P-CN	phosphoric acid and cyanuric acid–melamine complex (12:1), thermal condensation	24 times	[163]
O-CN	H_2_SO and HNO_3_, chemical oxidation	17 times	[164]
B-CN	ammoniotrihydroborate(H_3_NBH_3_), one-pot thermal polycondensation	12 times	[166]

**Table 4:** The performance and stability of different photocatalysts.

Photocatalyst	HER (μmol∙g^−1^∙h^−1^)	OER (μmol∙g^−1^∙h^−1^)	Stability (h/Times)	Ref.
MnO_x_/g-C_3_N_4_/CdS/Pt	1303.4	641.6	24 h (6 times) cycles	[194]
Pt/Ti-MOF-NH_2_	11.7	—	9 h (3 times) cycles	[72]
Ni-CdS	16,700	—	80–220 h	[182]
LaTiO_2_N/Sn_3_O_4_	887	—	18 h (3 times) cycles	[190]
BiVO_4_/g-C_3_N_4_	15.6	7.3	20 h (5 times) cycles	[220]
CdSe/P-g-C_3_N_4_	113.0	55.5	42 h (6 times) cycles	[221]
BiFeO_3_/g-C_3_N_4_	160.8	80.1	15 h (3 times) cycles	[222]
Co_3_(PO_4_)_2_/g-C_3_N_4_	375.6	177.4	12 h (4 times) cycles	[223]
P-g-C_3_N_4_/Ti_3_C_2_	627.1	305.4	40 h (8 times) cycles	[224]
Au-NiO_x_/TiO_2_	5.5	2.7	35 h (3 times) cycles	[225]
NiO-SrTiO_3_	28	—	24 h	[226]
Co-Pi/Bi-La_2_Ti_2_O_7_/Pt	66.6	32.1	7.5 h (3 times) cycles	[227]
Co_2_P/CdIn_2_S_4_	471.9	—	15 h (3 times) cycles	[191]
GaFeO_3_	9.0	4.5	12 h	[228]
NiO/NaTaO_3_: La	5900	2900	12 h (4 times) cycles	[229]
BiVO_4_-Ru/SrTiO_3_: Rh	40.1	18.6	11 h	[230]
Pt/CdS@Al_2_O_3_	62.1	—	30 h (10 times) cycles	[231]
Pt-loaded Mg/TiO_2_	850	425	30 h	[232]
RuO_2_/GaN:ZnO	1000	200	15 h (3 times) cycles	[55]
ZnCdS@DBTg-C_3_N_4_	8.87	—	12 h (4 times) cycles	[193]
O-CN/g-C_3_N_4_	6.97	—	25 h (5 times) cycles	[192]

**Table 5:** Catalytic properties and stability of some electrocatalytic materials.

Electrocatalyst	HER Overpotential (10 mA cm^−2^)	HER Exchange Current Density	Stability (h/cycles)	Surface Area (or ECSA)	Tafel Slope (mVdec^−1^)	Ref.
Mo_2_C@2D-NPCs	45 mV	0.0014 A cm^−2^	20 h	110.2 m^2^∙g^−1^	46	[235]
WC@NC	141 mV	0.78 A cm^−2^	Over 20 h	308.4 m^2^∙g^−1^	78.7	[236]
P-Mo_2_C/Ti_3_C_2_@NC	177 mV	—	60 h	20.4 mF∙cm^−2^	57.3	[237]
2D Mo_2_C/G	236 mV	—	1000 cycles	—	73	[238]
3D graphene foam	—	—	20,000 cycles	980 m^2^∙g^−1^	—	[239]
N, P-doped Mo_2_C@C	47 mV	2.042 mA cm^−2^	1000 cycles	156 m^2^∙g^−1^	71	[240]
Ni_2_P@NPCNFs	63.2 mV	—	3000 cycles	520 m^2^∙g^−1^	56.7	[241]
Mo_2_C@C@Pt	47 mV	—	1000 cycles	128.4 m^2^∙g^−1^	28	[242]
Ru-CoP/NCs	22 mV	—	20 h	178 m^2^∙g^−1^	56	[243]
β-Mo_2_C/N, P	181 mV	0.015 mA cm^−2^	2000 cycles	9.83 m^2^∙g^−1^	65.3	[244]
3D NiCo_2_O_4_@graphene	—	—	10,000 cycles	194.5 m^2^∙g^−1^	—	[245]

**Table 6:** Economic comparison of various hydrogen production technologies.

Technologies	Main Cost Factor	Advantages	Disadvantages	Application Phase
Electrocatalysis	Equipment, Electricity, Electrode material	Environmentally friendly, High purity	Low service life, High costs of noble metal catalyst	Initial commercialization
Photocatalysis	Efficiency, Photocatalytic material	Simple device, Low cost, Solar power, Clean energy	Poor efficiency	Laboratory
Fossil fuel	Raw material, Equipment, Carbon emissions	Mature and low cost 6.8–12 RMB/kg (Coal) 16–24 RMB/kg (Coal + CCS) 8–16 RMB/kg (Natural gas)	Carbon emissions 10–19 kg (CO_2_)/kg (H_2_) for coal and natural gas	Mature industrial application
Biomass	Raw material, Efficiency, Equipment	Renewable, Rich source, Clean	Impure product	Preliminary industrial demonstration
Waste	Product purity; Processing	Renewable, Resource recycling	Not mature	Preliminary industrial demonstration

## Data Availability

No new data were created or analyzed in this study. Data sharing is not applicable to this article.
